# Hypoxia signaling in cancer: Implications for therapeutic interventions

**DOI:** 10.1002/mco2.203

**Published:** 2023-01-23

**Authors:** Yan Zhuang, Kua Liu, Qinyu He, Xiaosong Gu, Chunping Jiang, Junhua Wu

**Affiliations:** ^1^ State Key Laboratory of Pharmaceutical Biotechnology National Institute of Healthcare Data Science at Nanjing University Jiangsu Key Laboratory of Molecular Medicine Medicine Medical School of Nanjing University Nanjing University Nanjing China; ^2^ Microecological, Regenerative and Microfabrication Technical Platform for Biomedicine and Tissue Engineering Jinan Microecological Biomedicine Shandong Laboratory Jinan City China

**Keywords:** hyperoxia, hypoxia‐inducible factor (HIF), immunotherapy, targeted theraphy, tumor hypoxia

## Abstract

Hypoxia is a persistent physiological feature of many different solid tumors and a key driver of malignancy, and in recent years, it has been recognized as an important target for cancer therapy. Hypoxia occurs in the majority of solid tumors due to a poor vascular oxygen supply that is not sufficient to meet the needs of rapidly proliferating cancer cells. A hypoxic tumor microenvironment (TME) can reduce the effectiveness of other tumor therapies, such as radiotherapy, chemotherapy, and immunotherapy. In this review, we discuss the critical role of hypoxia in tumor development, including tumor metabolism, tumor immunity, and tumor angiogenesis. The treatment methods for hypoxic TME are summarized, including hypoxia‐targeted therapy and improving oxygenation by alleviating tumor hypoxia itself. Hyperoxia therapy can be used to improve tissue oxygen partial pressure and relieve tumor hypoxia. We focus on the underlying mechanisms of hyperoxia and their impact on current cancer therapies and discuss the prospects of hyperoxia therapy in cancer treatment.

## Graphical abstract

Solid tumors gradually form a hypoxic malignant microenvironment during growth, which promotes tumor proliferation and metastasis by affecting metabolism, angiogenesis, and immune escape, and finally leads to therapeutic resistance. In recent years, hypoxia‐inducible factor inhibitors, direct targeting of hypoxic cells, and respiratory hyperoxia have been widely used to improve the hypoxic microenvironment of tumors and enhance the sensitivity of tumor therapy.

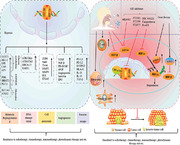

## 1 INTRODUCTION

Oxygen is an indispensable element for aerobic metabolism in human cells.^1^ Therefore, hypoxia often occurs in acute and chronic vascular diseases, pulmonary diseases, and cancer.^2^ At the periphery of a tumor, 70–150 μm away from the tumor blood vessels, the available oxygen is consumed by rapidly proliferating cells, which limits the diffusion of oxygen into the deep layers of the tumor, resulting in oxygen tension below 10 mmHg, known as tumor hypoxia.^3^ This harsh microenvironment is usually found in the core of most solid tumors, and the cause is related to the malformation of the vasculature.^4^ Compared with normal tissues, one of the most prominent hallmarks of solid tumors is the structure of their vascular network, and the vasculature cannot provide essential oxygen and nutrients for the growing tumor.^5^ Solid tumor hypoxia is typically classified into two subtypes, acute or chronic hypoxia. These two subtypes can lead to completely different hypoxia‐related responses, which may directly affect tumor development and response to treatment.^6^ Studies have found that tumor cells induce hypoxia through multiple mechanisms, such as a high metabolic rate^7^, ^8^ and high‐rate oxygen consumption,^9^, ^10^ thereby causing endothelial dysfunction or destroying oxygen delivery due to various effects on blood vessels.^11^ The formation of a chronic hypoxic environment activates the signaling pathway of hypoxia‐inducible factors (HIFs), accelerates tumor growth, improves tumor invasiveness, and promotes tumor metastasis.^12^ Researchers have cultured cancer cells in normal and hypoxic environments and found that the proliferation, invasion, and metastasis activities of cells are significantly higher in hypoxic environments than in normal oxygen‐containing environments.^13^, ^14^

For more than a century, extensive efforts have been made to address tumor hypoxia in both experimental and clinical settings.^15^ The key is to develop therapies that aim to target the hypoxic environment and improve intratumoral oxygenation to inhibit tumor progression and treatment resistance. To target hypoxic cells, inhibitors of hypoxia‐related pathways, such as HIFs and mammalian target of rapamycin (mTOR), have been developed.^16^ Researchers have also found ways to improve tumor oxygenation and relieve the hypoxic tumor environment, such as supplemental hyperoxia therapy.^17^ Oxygen therapy is one of the most widely used therapies in modern medicine and is also an intervention to relieve tissue hypoxia in critically ill patients.^18^ Here, we will summarize the role of hypoxia in tumor development, ways to target tumor hypoxia, ways to improve tumor oxygenation, the application of hyperoxia in tumor therapy and the impact of hyperoxia on currently available therapies.

## 2 THE ROLE OF HYPOXIA IN TUMOR PROGRESSION

Many tumors can survive under hypoxic conditions, mainly due to the expression of HIF in tumor cells.^19^ HIF was discovered serendipitously while studying the mechanism of erythropoietin (EPO) gene regulation.^20^ HIF‐1 is an isoform consisting of one of three alpha subunits (HIF‐1α, HIF‐2α, or HIF‐3α) and a beta subunit (HIF‐1β, also known as aryl hydrocarbon nuclear transporter, or ARNT).^21^ HIF‐1α is considered to be a master transcriptional regulator of the tumor cell response to hypoxia.^22^ Jiang et al. exposed human cervical cancer HeLa cells to O_2_ concentrations ranging from 0.125% to 20% and then analyzed HIF‐1 expression as a function of intracellular O_2_ concentration. The HIF‐1 DNA‐binding activity of cells and the concentrations of HIF‐1α and HIF‐1β proteins increased exponentially with decreasing O_2_ concentration.^23^

**FIGURE 1:**
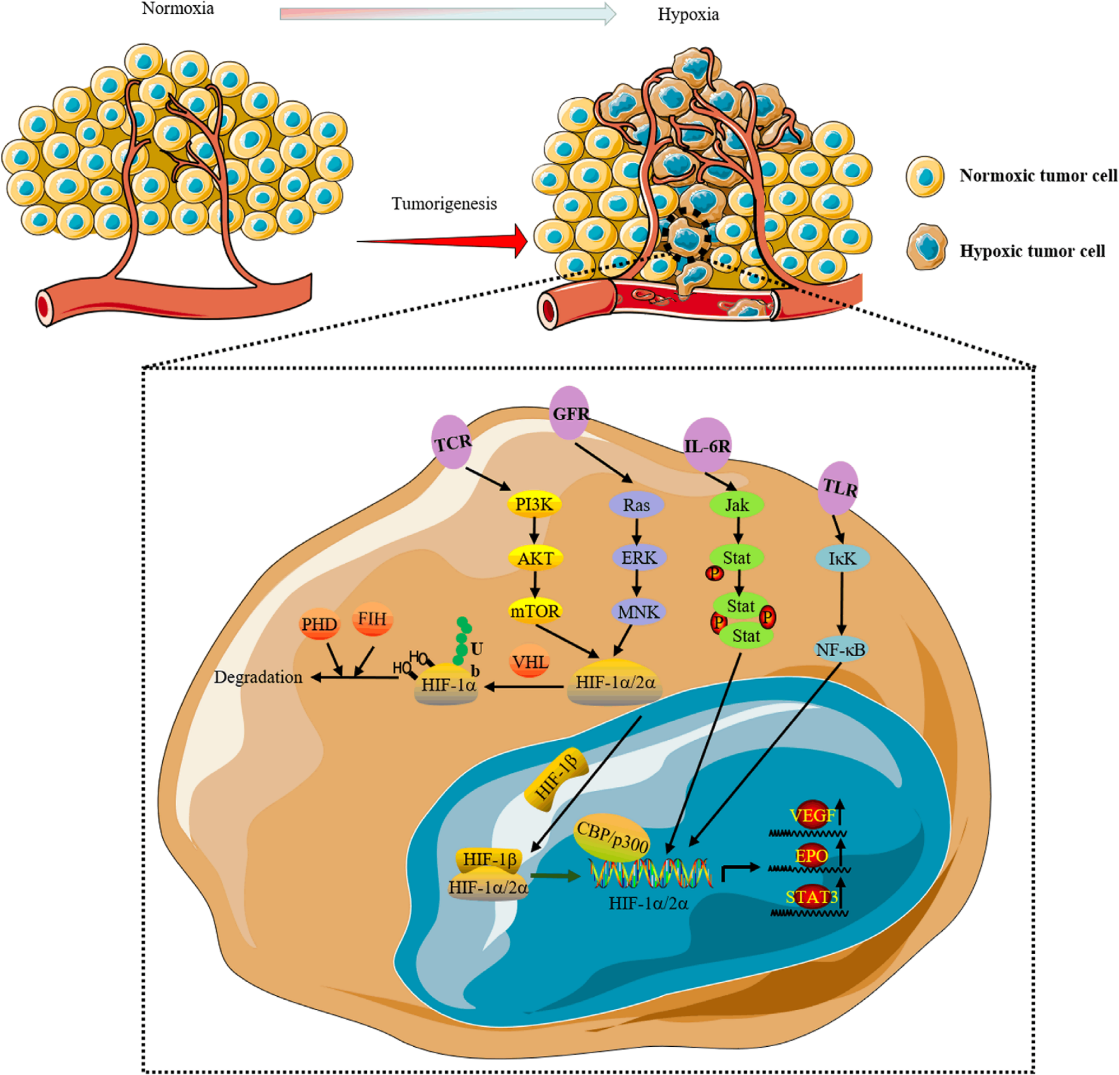
In the hypoxic tumor microenvironment, hypoxia‐inducible factor (HIF)‐1α is activated by a variety of receptors (such as TCR, GFR, IL‐6R, and TLR) on the plasma membrane through the mammalian target of rapamycin (mTOR), NF‐κB and JAK‐STAT signaling pathways. The accumulated HIF‐1α combines with HIF‐1β to form a dimer in the nucleus. CBP/P300 is a transcription cofactor, thus forming the HIF complex that initiates the transcriptional activity. The promotion of the transcription of multiple genes, such as vascular endothelial growth factor (VEGF), erythropoietin (EPO), and STAT3, is involved in cell adaptation to hypoxic stress. At the same time, HIF is degraded by proline hydroxylase (PHD), which is recognized by the ubiquitin ligase Von Hippel‒Lindau (VHL) for ubiquitination. Factors that inhibit HIF (FIH) can also suppress HIF‐1 expression by inhibiting its transcriptional activity

Von Hippel‒Lindau (VHL) syndrome is a rare autosomal dominant genetic disorder caused by pathogenic variants of the VHL gene. Approximately 70% of patients with VHL develop renal cell cancer (RCC) during their lifetime. The VHL protein acts as an E3 ubiquitin ligase and can ubiquitinate the HIF‐α subunit in an oxygen‐dependent manner, resulting in the proteolysis of HIF. VHL gene mutations lead to the stability of HIF subunits and the constitutive activation of HIF‐mediated transcriptional pathways in patients with VHL, causing the progression of renal cell carcinoma.^24^, ^25^, ^26^ In VHL‐deficient renal clear cell carcinoma, aberrant activation of Akt (also known as protein kinase B, PKB) in a HIF‐independent and prolyl hydroxylation‐dependent manner is one of the underlying drivers of renal carcinogenesis and metastasis.^27^ Ubiquitin‐specific protease 22 (USP22) can promote hypoxia‐induced hepatocellular carcinoma (HCC) stemness and glycolysis by deubiquitinating and stabilizing HIF1α.^28^ As oxygen levels decline, HIF‐α escapes degradation by VHL and accumulates. The activation capacity of HIF‐α is controlled by an asparagine hydroxylase (factors that inhibit HIF, FIH) that acts on the C‐terminal domain (C‐TAD) of the HIF‐1α protein. Inhibiting FIH activity under hypoxia is necessary and sufficient for activating HIF‐1α c‐TAD activity.^29^ In hypoxic brain tumor cells, an inhibitor of DNA binding protein 2 (ID2) binds to and disrupts the VHL complex, thereby preventing ubiquitin‐mediated proteasomal degradation of HIF‐2α.^30^ Hypoxia‐induced upregulation of HIF‐1 also leads to the activation of vascular endothelial growth factor (VEGF). Chiarotto used different oxygen concentrations to verify the upregulation of VEGF mRNA in three cervical cancer cell lines (O_2_ concentrations of 21%, 6.25%, 4.85%, 3.46%, 2.11%, 1.57%, 1.00%, and 0%), demonstrating that the presence of VEGF mRNA may be a marker of hypoxia.^31^ Takahiro and co‐workers also demonstrated that HIF‐1α was first produced in metastatic lymph nodes under hypoxia, leading to high expression of VEGF‐A and then increased expression of VEGF‐C. VEGF is highly expressed in most human cancer cell types, and hypoxia has been shown to be a major inducer of VEGF gene transcription (Figure 1).^32^, ^33^

Rapid uncontrolled growth of tumor cells, inadequate blood supply, and hypoxia are typical microenvironmental features of most solid tumors.^34^ Cancer cells rewire their metabolic programs, initiate angiogenesis and metastasis, influence immune responses, and launch rapid DNA damage repair responses to sustain growth and survival.^16^ Next, we outline the effects of hypoxia on tumor behavior and the intrinsic mechanisms, underscoring the role of HIF‐mediated cell metabolism and immune responses.

### 2.1 Tumor hypoxia induces abnormal cell metabolism

The role of oxygen in the metabolic pathway of cells is paramount because cancer cells reside in nutrient‐ and oxygen‐deficient environments due to the rapid growth of tumors.^35^ To maintain proliferation, cancer cells reconnect their metabolic pathways and find another way to maintain their own growth. Metabolic adaptation is a new feature of cancer.^36^, ^37^ This metabolic reprogramming is known as the Warburg effect in cancer cells and is mediated by HIF‐1.^38^, ^39^, ^40^ Alterations in gene regulatory networks can drive the heterogeneous behavior of cancer metabolism.^41^ Although some mutations have been identified, such as isocitrate dehydrogenase‐1 (IDH1) and IDH2,^42^, ^43^ mutations are only one of the reasons why hypoxia alters tumor metabolic pathways. Oxygen, glucose, and glutamine in the tumor microenvironment (TME) are considered to be the main nutrients that promote cell growth.^44^, ^45^ Hypoxia can provide energy to tumor cells by activating transcriptional programs that induce glucose uptake and glycolysis.^46^, ^47^ Tumor cells adaptively turn to androgen/androgen receptor‐independent pathways to survive and grow, resulting in therapeutic resistance.^48^ This is mediated by glucose‐6‐phosphate isomerase (GPI), which is transcriptionally repressed by androgen receptors in response to hypoxia but recovers when androgen receptors are inhibited. GPI maintains glucose metabolism and energy homeostasis during hypoxia by redirecting glucose to hypoxia‐induced glycolytic pathways.^49^ In response to hypoxia, pancreatic ductal cancer cells expressing activated Kirsten rat sarcoma viral oncogene homolog (KRAS) increase their expression of carbonic anhydrase 9 (CA9), which regulates cellular pH and is directly induced by hypoxia, by stabilizing HIF‐1α and HIF‐2α, thereby regulating pH and glycolysis.^50^ The All1‐fused gene from chromosome 9 (AF9) is upregulated in HCC to recognize acetylated cMyc and promote the expression of hypoxia tolerance and glycolytic genes by forming a complex with HIF1α.^51^ Macropinocytosis is an endocytic process caused by membrane folds, leading to phagocytosis of extracellular fluid (proteins), digestion of proteins, and subsequent incorporation into the cell biomass. Hypoxia supports macropinocytosis‐mediated cancer metabolic reprogramming through the HIF/EHD2 (EH dome‐containing protein 2) pathway and uses extracellular proteins as a nutrient for survival.^52^

Glutamine is the most abundant amino acid in the blood and is critical for the synthesis of tricarboxylic acid cycle metabolites, nonessential amino acids, and adenosine triphosphate (ATP) energy, on which cancer cells and rapidly proliferating cells depend.^53^, ^54^, ^55^ HIF‐2α‐induced expression of the glutamine transporter solute carrier family 1, member 5 (SLC1A5) mediates glutamine‐induced ATP production and glutathione synthesis and renders pancreatic cancer resistant to gemcitabine.^56^ Glucose delivery is impeded in hypoxic regions of the body, which increases the cell's dependence on other carbon sources, such as L‐glutamine. Autophagy‐related 12 (ATG12) deficiency leads to decreased intracellular L‐glutamine levels during hypoxia, while cancer cell killing increases after L‐glutamine depletion. Tumor microenvironment analysis showed that ATG12 targeting resulted in a decreased tolerance to hypoxia, increased necrosis, and sensitivity to treatment. This suggests that ATG12 plays an essential role in maintaining L‐glutamine homeostasis.^57^

Cell metabolism mainly refers to the metabolism of energy and substances. It is widely known that mitochondria are the energy reservoir in organisms. Many studies have focused on whether HIF regulates metabolic pathways that influence mitochondrial viability.^58^, ^59^ Mitochondrial serine hydroxymethyltransferase (SHMT2) is required for cancer cells to adapt to the tumor environment.^60^ Jiangbin Ye proposed that HIF‐1 induces SHMT2 under hypoxia and that this induction is most pronounced in cells overexpressing the oncogenic transcription factor Myc. When these cells are hypoxic, they require SHMT2 expression to sustain the cellular NADPH/NADP^+^ ratio. Loss of SHMT2 in hypoxic cells increases reactive oxygen species (ROS) levels, resulting in cell death.^61^ Inhibition of phospholipase C under hypoxia promotes the metabolic process of cancer cells, reduces the formation of mitochondrial ROS, and converts tumor bioenergy into glycolysis by inhibiting Ca2^+^ entry into mitochondria.^62^ Collectively, the metabolic reprogramming associated with cancer has received much attention, and how cancer cells meet these needs in vivo is increasingly being studied. However, considering that the hypoxic TME and its interaction with metabolic processes are highly complex, tumor metabolism needs to be comprehensively evaluated. Hypoxia and tumor metabolism still need to be explored.

### 2.2 Tumor hypoxia promotes angiogenesis

In 1971, Professor Folkman proposed the theory that “tumor growth and metastasis depend on neovascularization”, which provided a new research direction and theoretical rationale for antiangiogenic antitumor drugs.^63^, ^64^ The mechanism of angiogenesis is complex, and there are many factors involved in promoting angiogenesis. More than 40 molecules have been identified to play a key role in vascular recruitment.^65^ These include VEGF, transforming growth factor‐β (TGF‐β), platelet‐derived endothelial cell growth factor (PD‐ECGF), angiopoietin, HIF‐1, survivin, and EPO.^66^, ^67^, ^68^, ^69^, ^70^, ^71^ However, to date, most studies have focused on HIF and VEGF and their receptors. In the hypoxic environment of the internal tumor mass, the HIF‐1 dimeric complex remains stable and activates the expression of numerous genes responsible for the angiogenesis process.^71^ HIF‐1‐induced proteins include VEGF, which promotes vascular permeability, and basic fibroblast growth factor (bFGF), which promotes endothelial cell growth.^72^, ^73^ Because of the essential function of angiogenesis in tumor development, the development of antiangiogenic drugs has been put on the agenda. The commonly used antiangiogenic drugs are bevacizumab, ramucirumab, and olaratumab, among others.^74^, ^75^, ^76^, ^77^ Traditional angiogenesis inhibitors mainly block VEGF signaling and prune rather than eradicate tumor vessels.^78^ The expected antiangiogenic therapy has not been achieved thus far because these therapies are less effective against tumors with alternate blood supply sources. Angiogenesis inhibitors may be more beneficial in combination with other conventional chemotherapy agents.^79^, ^80^, ^81^

### 2.3 Tumor hypoxia promotes metastasis

Hypoxia is associated with metastasis; however, how it affects metastasis progression is controversial. Currently, epithelial‐mesenchymal transition (EMT) has mostly been studied in relation to hypoxia and metastasis. EMT is a key process in the metastasis and colonization of cancer cells from the primary tumor to distant organs. Hypoxia plays an important role in triggering EMT by regulating HIFs.^82^, ^83^, ^84^ Studies have demonstrated that HIF has a direct regulatory effect on EMT‐related proteins, such as zinc finger E‐box binding homeobox 1, Snail and Twist.^85^, ^86^, ^87^ At the same time, HIF can also modulate microRNA (miRNA) to promote the cellular EMT process.^88^, ^89^, ^90^, ^91^ Many studies have reported the downregulation of drosha and dicer, two key enzymes involved in miRNA biogenesis.^92^, ^93^ Dicer is a key enzyme in miRNA maturation. HIF‐1α downregulates the expression of dicer by promoting ubiquitination of the E3 ligase parkin, which further inhibits known tumor suppressors, such as the microRNAs let‐7 and microRNA‐200B. Downregulation of dicer expression can significantly enhance cancer metastasis through microRNA‐200.^94^, ^95^ Hypoxia can promote EMT in tumor‐bearing mice by downregulating miRNA expression.^96^ Recent studies have shown that CSN8, the smallest and most conserved subunit of the COP9 signalosome (CSN), is a key regulator of hypoxia‐induced EMT and dormancy, which endows colorectal cancer (CRC) cells with stronger invasion and metastasis abilities.^97^

### 2.4 Tumor hypoxia accelerates DNA damage repair

Some cancer therapies kill tumor cells by causing DNA damage; however, cancer cells have been shown to activate a variety of repair mechanisms and signaling pathways as a response to overcome DNA damage.^98^ The repaired cancer cells then become more resistant to standard treatment. The success of cancer treatment is limited by two factors: insufficient DNA damage during treatment and rapid DNA repair after treatment.^99^ The ATM and RAD3‐related (ATR)/Chk1 and ataxia‐telangiectasia mutation (ATM)/Chk2 signaling pathways play key roles in the DNA damage response.^100^ ATR/Chk1 signaling is usually activated by single‐stranded DNA or large‐volume DNA lesions, whereas the ATM/Chk2 pathway is activated by DNA double‐strand breaks.^101^ After DNA damage caused by standard treatment, tumor hypoxia can drive DNA repair to allow tumor cells to continue to grow, giving rise to resistance to antitumor therapy.^102^ The antiangiogenic agent cediranib inhibits the expression of the homology‐directed DNA repair (HDR) factor BRCA1/2 and RAD51 recombinase and promotes the sensitivity of the poly (ADP‐ribose) polymerase (PARP) inhibitor olaparib. The combination of cediranib and olaparib induces lethality by suppressing DNA repair. The activity of sildiranib as a DNA repair inhibitor has expanded its clinical utility.^103^ There is a growing body of evidence that combination treatment with drugs with the ability to inhibit HDR (e.g., androgen receptor inhibitors) can modulate DNA repair,^104^, ^105^ with potential implications for the development of combination therapies aimed at exploiting DNA repair defects induced in cancer cells.

### 2.5 Tumor hypoxia promotes immune escape

Aside from obtaining an adequate energy supply, tumor occurrence and development must evade various immune surveillance mechanisms of the body.^106^ The hypoxic TME can directly affect the biology of infiltrating immune cells and the response to therapy. (1) Hypoxia can directly upregulate the expression of various cellular immune checkpoints (such as programmed cell death‐ligand 1, PD‐L1) in the TME.^107^, ^108^, ^109^ HIF‐1α interacts with histone deacetylase 1 (HDAC1) and simultaneously relies on polycomb repressive complex 2 protein with histone methyltransferase activity to induce chromatin remodeling, thereby leading to immune dysfunction. Hypoxic effects on immune cells also lead to insufficient PD‐L1 and PD‐L2 responses.^110^ Targeting HIF‐1α with drugs or genes can inhibit PD‐L1 expression in the TME and enhance interferon production by T cells.^111^, ^112^, ^113^ Targeting HIF‐1α reverses immune dysfunction and overcomes resistance to PD‐L1 blockade.^114^ (2) Hypoxia maintains the function of myeloid‐derived suppressor cells (MDSCs), which have immunosuppressive activity, allowing cancer cells to escape immune surveillance and resist immune checkpoint blockade.^115^, ^116^ HIF‐1 induces extracellular nucleoside triphosphate diphosphate hydrolase 2 (ENTPD2/CD39L1) overexpression in hepatocellular carcinoma clinical specimens. ENTPD2 converts extracellular ATP to 5‐AMP, which prevents the differentiation of MDSCs and therefore promotes the maintenance of MDSCs.^117^ HIFs can also activate the transcription of chemokine (C‐C motif) ligand 26 in cancer cells and recruit MDSCs expressing chemokine (C‐X3‐C motif) receptor 1 to primary tumors.^118^ This suggests that targeting MDSC recruitment is an attractive approach for the treatment of solid tumors. (3) HIFs can also switch tumor‐associated macrophages (TAMs) from an antitumorigenic (M1‐like) phenotype to a protumorigenic (M2‐like) phenotype.^119^, ^120^, ^121^ Mechanistically, HIF‐1α‐stabilizing long noncoding RNA (HISLA) can block the hydroxylation of HIF‐1α and inhibit the degradation of HIF‐1α by proline hydroxylase 2 (PHD 2). Simultaneously, lactate released from tumor cells upregulates HISLA in macrophages, and blocking the transmission of HISLA in vivo can inhibit drug resistance in breast cancer.^122^ HIF‐1α can also induce TAMs to secrete IL‐23, which activates the proliferation of regulatory T cells (Tregs), promotes the expression of IL‐10 and TGF‐β, and thus inhibits the capacity of cytotoxic lymphocytes to kill tumor cells.^123^ Specific deletion of HIF‐2, but not HIF‐1, in cancer‐associated fibroblasts, improve pancreatic cancer patient survival by reducing the recruitment of immunosuppressive macrophages. Treatment with the clinical HIF‐2 inhibitor PT2399 significantly reduced macrophage chemotaxis and M2 polarization in vitro.^124^ (4) Does hypoxia inhibit T‐cell function? The effects of hypoxia on T cells are controversial. Some findings have indicated that O_2_ is required for T‐cell activation and the development of effector functions and that HIF‐1α acts as a negative regulator of T‐cell responses.^125^ For example, tumor hypoxia activates the γδT‐cell protein kinase A pathway at the transcriptional level, resulting in the inhibition of the activating receptor natural killer cell group 2D (NKG2D). Alleviating tumor hypoxia enhanced the expression of NKG2D and enhanced the antitumor function of γδT cells.^126^ The combination of the hypoxia‐activated prodrug TH‐302 and checkpoint blockade of PD‐L1 or cytotoxic T lymphocyte‐associated antigen‐4 can restore the killing function of T cells, and the tumor cure rate was more than 80%.^127^ Hypoxia downregulates nuclear receptor coactivator 3, an epigenetic factor that maintains the basic level of cGMP‐AMP synthase (cGAS) expression, which leads to the inhibition of cGAS, thereby inhibiting the function of T cells.^128^ Other studies using CD8+ T cells during resting periods of hypoxia or VHL‐deficient cells have reported that HIF‐1α signaling increases T‐cell function.^129^, ^130^, ^131^ For example, 2‐hydroxyglutarate (2‐HG) is produced by CD8+ T lymphocytes in response to T‐cell receptor triggering and environmental hypoxia, and 2‐HG significantly boosts the proliferation, persistence, and antitumor capacity of CD8+ T lymphocytes in vivo.^132^ VEGF‐A production by tumor cells is associated with a poor prognosis; however, deletion of the HIF target gene VEGF‐A in CD8+ T cells accelerated tumorigenesis and altered angiogenesis. The deletion of T‐cell‐specific genes demonstrated that HIF‐1α is an important regulator of T‐cell effector responses in the TME. Therefore, VEGF‐A in effector CD8+ T cells contributes to T‐cell infiltration.^133^

## 3 HYPOXIA‐TARGETED THERAPEUTICS IN CANCER

Since the discovery of the hypoxic tumor environment, sensitization of hypoxic cells in tumors and improvement of the hypoxic tumor environment have been the focus of tumor research.^134^, ^135^, ^136^ Hypoxia is arguably one of the most intriguing therapeutic targets for cancer.^137^, ^138^ The primary strategies for treating hypoxic tumors are to release hypoxia‐targeted drugs and improve the hypoxic environment. In this subsection, we review hypoxia‐targeted tumor therapy methods. Multiple targeting pathways have been proposed for hypoxic tumor cells, including specific targeting of HIFs,^139^, ^140^, ^141^ gene therapy,^142^, ^143^, ^144^ targeting hypoxic tumors, and targeting pathways that have important effects on hypoxic cells, such as the mTOR pathway.^145^, ^146^ In addition, because tumor tissue has a lower pH than healthy tissues, this special characteristic can be used to target acids to induce tumor death.^147^, ^148^, ^149^

### 3.1 HIF inhibitors

HIFs are significantly upregulated in many cancer types, and the application of HIF inhibitors as anticancer therapies has captured great interest.^150^ The oxygen‐dependent activity of HIFs is mainly transmitted by HIF‐α subunits. In human cells, there are three HIF‐α subtypes, among which HIF‐1α and HIF‐2α are the most widely studied. The first inhibitor of HIF‐1 discovered was camptothecin, which inhibits HIF‐1 through a mechanism that blocks protein accumulation.^151^ Subsequently, a novel tricyclic carboxamide inhibitor of HIF‐1α, NSC 644221, was developed. NSC 644221 inhibits HIF‐1α protein expression in a time‐ and dose‐dependent manner. However, it does not inhibit HIF‐1β protein expression.^152^ Px‐478, which has entered clinical stage I, plays an antitumor role by inhibiting HIF‐1α deubiquitination, leading to polyubiquitinated HIF‐1α degradation.^153^, ^154^ HIF‐2α contains the Per‐Arnt‐Sim B (PAS‐B) domain, and this particular PAS domain contains a relatively large cavity that can be occupied by water or small molecules, including PT2385.^155^, ^156^ PT2385 is a HIF‐2α antagonist that inhibits the expression of HIF‐2α by disrupting HIF‐2α/HIF‐1β (ARNT) heterodimerization. PT2385 has passed phase I clinical trials.^140^, ^157^ Belzutifan (MK‐6482) is a second‐generation small‐molecule HIF‐2α inhibitor that outperforms the pharmacological properties of the first‐generation compound PT2385 and has shown efficacy and safety in phase III trials in patients with advanced clear cell renal cell carcinoma.^24^, ^72^, ^158^ James Brugarolas designed a selective HIF‐2α antagonist, PT2399, based on the structure of HIF‐2α PAS‐B; PT2399 selectively disrupts the heterodimerization of HIF‐2α and HIF‐1β.^159^ Many compounds have been shown to antagonize HIF activity, including echinomycin,^160^ 17‐allylaminogeldanamycin,^161^ acriflavine,^162^ and the thioredoxin redox inhibitors 1‐methylpropyl 2‐imidazolyl disulfide^163^ and 3‐(5′‐hydroxymethyl‐2′‐furyl)‐1‐benzylindazole (YC‐1).^164^ The anticancer effect of these drugs is likely due to HIF inhibition; however, these drugs do not specifically target HIFs. Meanwhile, many of these compounds cannot be used as HIF inhibitors due to side effects or failure in clinical trials. Because HIFs are highly expressed not only in cancer cells but also in some normal tissues in some cases, it is unclear whether drugs targeting HIFs could have serious side effects in humans. Hopefully, clinical studies will provide an answer.

### 3.2 Gene therapy

Gene therapy is a promising strategy for the treatment of various genetic and acquired diseases. However, to minimize the side effects of gene therapy, gene expression must be targeted to specific tissues.^165^ HIFs accumulate in anoxic tissues and have three domains: the DNA binding domain, transcription activation domain, and oxygen‐dependent degradation (ODD) domain.^166^ An attractive strategy to inhibit HIF activity is to disrupt the HIF‐1α/P300 complex, as P300 is a key coactivator of hypoxia‐induced transcription. Blocking the interaction between HIF‐1 and the P300 region attenuates the expression of HIF‐1.^69^ The ODD domain contains proline residues that are recognized and hydroxylated by proline hydroxylase (PHD 1–3). Prolyl hydroxylation promotes HIF‐α binding to the VHL complex, resulting in chromosomal degradation of HIF proteins.^167^ IDH1 catalyzes the oxidative decarboxylation of isocitrate to α‐ketoglutaric acid (α‐KG). The stability of HIF‐1α is regulated by α‐KG, and increasing α‐KG reduces the expression of HIF‐1α.^168^ Gene therapy may be one of the most promising cancer treatment strategies. Tumorigenesis is the result of genetic alterations and accumulation. Therefore, intervention with gene therapy for these genetic alterations may alleviate tumor growth.

### 3.3 Targeting hypoxic tumors

Researchers have harnessed the properties of hypoxic cells, targeting them with hypoxia‐activated prodrugs (HAPs) or bioreductive agents.^169^ Appropriate prodrugs must diffuse efficiently to distant and hypoxic tumor regions and then specifically convert to the cytotoxic active form and diffuse over limited distances to kill adjacent hypoxic tumor cells.^170^ For example, Tirapazamine (TPZ) is a bioreduction‐activated drug among a hypoxia‐selective benzotriazine series of antitumor drugs.^171^ In a phase II trial of TPZ in combination with cisplatin and radiotherapy for advanced squamous cell carcinoma of the head and neck, TPZ enhanced the cytotoxicity of radiotherapy and cisplatin in the presence of hypoxic tumors.^172^ In addition, Vlahovic demonstrated that the phosphorylated platelet‐derived growth factor receptor β (PDGFR‐β) inhibitor imatinib, in addition to inhibiting p‐PDGFR‐β, can also downregulate VEGF and reduce intratumoral interstitial fluid pressure in lung adenocarcinoma.^173^ HAPs are important drugs for the treatment of hypoxic tumors because they have cytotoxic effects only in hypoxic environments and show reduced side effects in normal tissues under normoxic conditions. Although HAPs can kill hypoxic cells, insufficient tumor hypoxia limits their efficacy.

Multifunctional nanoparticles can integrate various key components, such as drugs, genes, and targeting ligands, using unique delivery platforms. Due to the enhanced permeability and retention effect (EPR effect),^174^ selective accumulation in solid tumors can be used to target hypoxic tumors more effectively.^175^ Bu et al. proposed a strategy of X‐ray‐induced nitrosation stress based on zeolitic imidazole framework‐82 with the assistance of polyvinyl pyrrolidone for the treatment of hypoxic prostate cancer. The proliferation of hypoxic prostate cancer cells was effectively inhibited by the continuous release of the hypoxic response ligand and Zn^2+^.^176^ Live photosynthetic bacteria (PSB) have been used as hypoxia‐targeting vectors for the treatment of hypoxic tumors due to their near‐infrared chemotaxis and facultative aerobic physiological properties. PSB “integrates” the characteristics of hypoxia‐targeting agents and photothermal therapeutic agents and can achieve hypoxia‐targeted cancer therapy without later modification.^177^ Lu integrated losartan (LOS), doxorubicin (DOX), and Fe ions into a system. LOS can reduce intratumoral collagen and alleviate tumor hypoxia. The Fenton reaction triggered by Fe ions promotes ROS production in tumors, and DOX enters tumors and inhibits tumor growth.^178^ E6 chloride loaded with anoxic amphipathic dendritic nanoparticles can target hypoxic tumors and activate dendritic cells, induce strong antigen‐specific immune memory effects, and prevent tumor metastasis and recurrence in vivo.^179^ Recently, a nanoparticle‐assisted immunometabolic therapy strategy targeting the ATP‐adenosine axis has been proposed to increase extracellular ATP levels while preventing the accumulation of immunosuppressive adenosine and alleviating hypoxia.^180^ We propose that HAPs can be used in combination with nanomedicine to obtain a mutually beneficial nanosystem. For example, HAPs have been used in combination with vascular‐disrupting agent (VDA) nanomaterials for the effective treatment of solid tumors. This approach selectively enhanced tumor hypoxia and TPZ in the treatment of metastatic 4T1 breast tumors.^171^ HAPs plus nanomedicine can be used for the synergistic treatment of tumors.

### 3.4 Others

Treatments targeting key components of tumor hypoxia signaling pathways, including mTOR, are one of the current approaches to cancer therapy.^181^, ^182^ mTOR activity promotes angiogenesis by regulating HIF‐1α transcription and translation.^183^ The mTOR inhibitor everolimus, which has been tested in phase III clinical trials, can prolong the progression‐free survival of patients with advanced renal cell carcinoma by inhibiting the promotion of HIF‐1 by mTOR.^184^ The dual inhibition of PI3K/mTOR by apitolisib did not have more effective antitumor activity than mTOR inhibition alone, possibly because the complete blockade of PI3K/mTOR signaling led to multiple adverse events. The beneficial effect of the mTOR inhibitor alone may be due to the inhibition of HIF‐1α.^185^ Of course, more experiments are needed to verify this hypothesis. Hypoxic tumor cells show reduced intracellular pH compared with normal cells because tumors exhibit elevated metabolism, an acidic pH, and nutrient and oxygen deficiencies.^2^, ^186^ Nanoparticles responsive to the acidic TME can target hypoxia and be used in cancer therapy. Layer‐by‐layer (LbL) nanoparticles exhibit a pH‐sensitive outer layer that has been shown to target and be retained in hypoxic tumor areas. The neutral layer is shed in acidic environments, exposing a surface of charged nanoparticles, which can be easily absorbed by tumor cells.^187^ Low oxygen and pH levels induce hyaluronic acid LbL nanoparticles to effectively target and penetrate hypoxic cells in vitro and in vivo.^188^

Hypoxic TME is still one of the major causes of resistance to radiotherapy, chemotherapy, and immunotherapy,^189^ and targeting hypoxic cells and improving tumor oxygenation have been a major focus of tumor therapy. In the above section, we introduced therapies targeting hypoxia. Next, we will focus on tumor therapies that can improve tumor oxygenation.

## 4 WAYS TO IMPROVE INTRATUMORAL OXYGENATION

**FIGURE 2:**
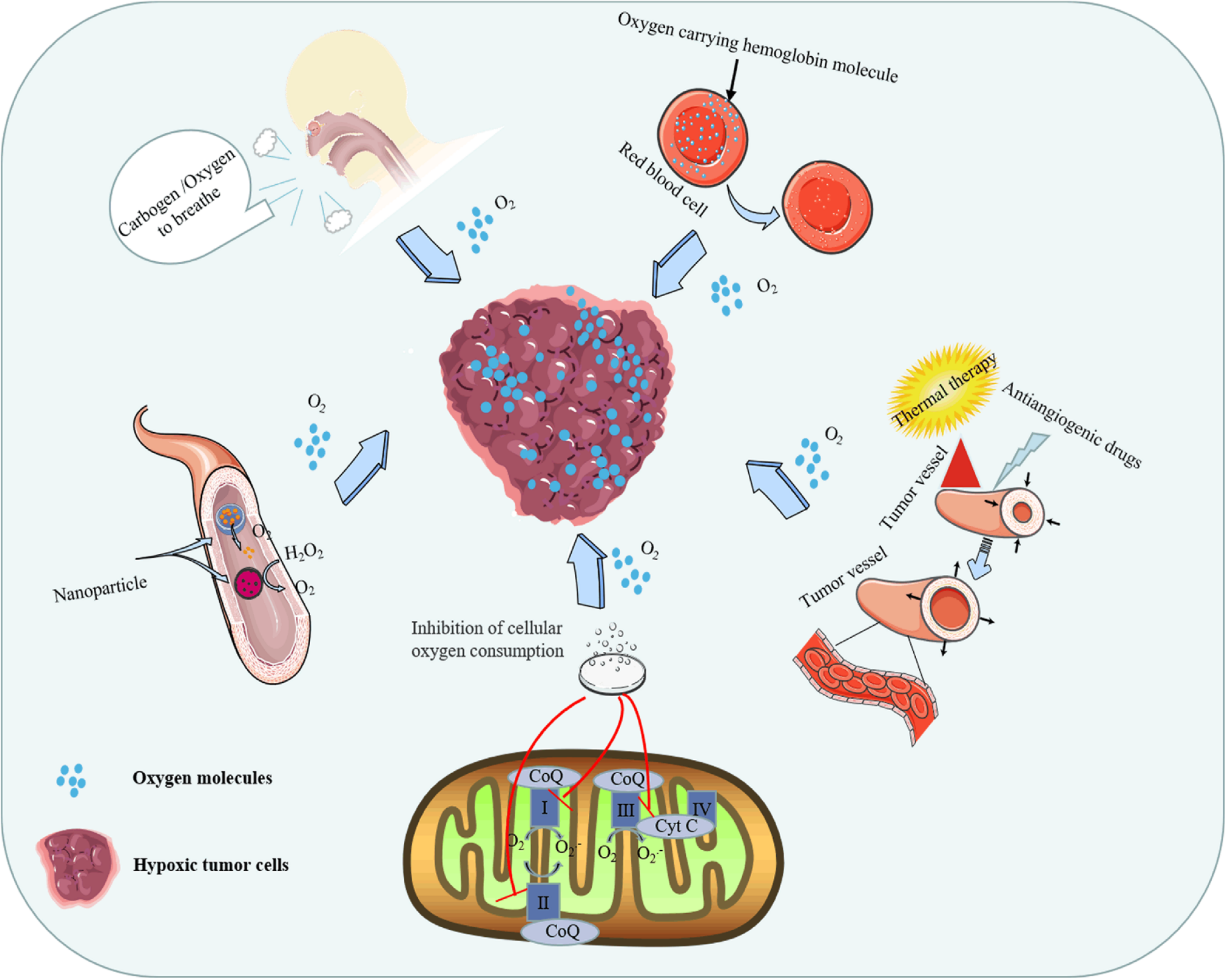
Schematic diagram of methods to improve tumor oxygenation. Tumor oxygenation improvement methods include the uptake of high‐concentration oxygen (normobaric/hyperbaric) or carbogen to increase vascular oxygen concentration, thereby increasing tumor oxygen partial pressure to reverse tumor hypoxia. Hemoglobin is an oxygen carrier that releases oxygen in tumor hypoxic tissue, and hyperthermia is used to increase blood flow by expanding blood vessels and leads to increases in oxygenation of the tumor. Antiangiogenic drugs normalize the overall function of tumor vessels to improve tumor blood supply and increase the tissue oxygen content. Drugs inhibit cellular respiration by inhibiting the oxidative respiratory chain, reducing the rate of oxygen consumption, and indirectly improving tumor oxygenation. Nanoparticles carry oxygen or catalyze the decomposition of H_2_O_2_ to obtain oxygen to relieve hypoxia in tumor tissue

To date, several methods have been proposed to increase the oxygen content of tumor tissue (Figure 2), including normobaric hyperoxia respiration,^190^ hyperbaric hyperoxic respiration,^191^ and carbogen inhalation^192^ to promote tumor oxygen partial pressure (pO_2_); nanomaterials to deliver/produce oxygen^193^, ^194^; hyperthermia to increase tumor oxygen by increasing blood flow within the tumor^195^; antihypoxic drugs to improve tumor oxygenation by killing hypoxic cells^196^; antiangiogenic drugs^197^; and promotion of oxygen accumulation by inhibiting tumor cell respiration.^198^

### 4.1 Respiratory hyperoxia at normobaric pressure enhances the pO_2_

For normobaric hyperoxic respiration, in a standard atmosphere with an oxygen content between 20% and 100%, mice were treated with 60% O_2_ indefinitely without signs of toxicity.^199^, ^200^ However, long‐term respiratory hyperoxia may have adverse effects on multiple organ systems exposed to O_2_ > 60%. Sustained hyperoxia (>60% oxygen) normally results in pulmonary and ocular toxicity due to oxidative damage to endothelial and epithelial cells.^201^ The initial use of hyperoxia therapy in cancer was to alleviate postoperative hypoxia in rectal and sigmoid cancers.^202^ Respiration with 100% O_2_ (1 h) alleviates hepatic hypoxia by inhibiting HIF‐2α expression.^203^ Multiple studies have been conducted showing that respiratory hyperoxia improves tumor oxygenation, which can have multiple beneficial effects as a cancer treatment modality.^204^, ^205^ Hyperoxia (60% O_2_) can also reverse the immunosuppressive TME and promote antitumor immunity.^206^

### 4.2 Hyperbaric hyperoxic respiration enhances the tumor pO_2_

Hyperbaric hyperoxic respiration treatment involves exposure to a high level of oxygen at a higher atmospheric pressure The oxygen pressure is much higher than at sea level. The pressure at sea level is defined as standard atmospheric (ATM) pressure, a value of 101.325 kPa, which can increase the oxygen tension of tissue or venous blood vessels, likely resulting in increased oxygen concentration. The specified criteria in hyperbaric oxygen treatment (HBOT) stipulate that patients are offered pure oxygen (100%) for 2–3 h under 1.5–2.5 absolute atmospheres (ATA).^207^ At present, respiratory hyperoxia is widely used as an adjuvant therapy for various pathological conditions, principally those related to hypoxia and/or ischemia.^208^ In triple‐negative breast cancer, HBO treatment can alleviate tumor hypoxia, thereby inhibiting tumor growth and metastasis.^209^ HBO can inhibit the hypoxia‐activated signal transducer and activator of transcription 3 (STAT3), slowing tumor growth and drug resistance.^210^ As early as 1968, Churchill‐Davidson used HBO to relieve hypoxia and promote radiotherapy.^211^ Based on clinical results, HBO has the effect of improving tumor oxygenation, and the use of HBO during radiotherapy has been proven to improve the radiation response of solid tumors.^212^ In addition, HBO can promote the efficacy of cytotoxic drugs.^213^, ^214^

### 4.3 Delivery of nanoparticle oxygen‐generating/oxygen‐carrying systems to alleviate tumor hypoxia

Various researchers have made substantial efforts devoted to the development of different nanocarriers in the hope of using nanotechnology to deliver oxygen to reverse tumor hypoxia and improve the efficacy of existing treatments.^194^, ^215^, ^216^ These methods can be simply divided into the following groups:

**Oxygen‐loaded nanocarriers**: Oxygen‐containing nanomaterials can directly deliver oxygen to hypoxic tissues and alleviate hypoxia. Researchers have developed a variety of nanomaterials with excellent oxygen‐carrying capacity, as shown by the following examples.

**Perfluorinated compounds to deliver oxygen**: Fluorine has low polarizability; thus, the low polarizability is converted to low intermolecular van der Waals forces, and van der Waals forces are the intermolecular forces that hold nonpolar molecules together. Accordingly, liquid perfluorocarbons behave close to ideal, gas‐like liquids.^217^, ^218^ Perfluorinated compounds are synthetic biologically inert compounds, and aqueous emulsions of fluorinated hydrocarbons are capable of dissolving large amounts of oxygen.^219^ The only perfluorochemical O_2_ carrier approved for clinical use by the Food and Drug Administration (FDA) is fluorocarbon‐DA (Fluosol‐DA), but it has been withdrawn from the market due to its cumbersome implementation and limited efficacy.^220^ Other perfluorochemical emulsions are being studied as oxygen transporters to alleviate tumor hypoxia.^221^, ^222^ The oxygen‐generating potential of several nanocomposite fluorine materials provides an extra strategy to enhance tumor oxygenation, supplemented by oxygen‐carrying nanocarriers to enhance tumor tissue oxygen levels and currently available therapies to overcome hypoxia‐related resistance and metastasis.^223^, ^224^

**Hemoglobin‐based oxygen carriers to deliver oxygen**: Hemoglobin‐based oxygen carriers (HBOCs) were originally developed to provide an alternative to blood transfusions. As the realization that hemoglobin solution is not only a substitute for erythrocytes but also has additional properties, including hemodynamic effects related to its osmotic pressure, nitric oxide scavenging effects, and effects on oxygen delivery and microcirculation, the broader concept of “hemoglobin therapy” was born.^225^ Because HBOCs can regulate oxygen delivery in the circulatory system, hemoglobin therapy is a promising oxygen therapy that can enhance the efficacy of other tumor therapies, such as radiotherapy and chemotherapy.^226^, ^227^, ^228^, ^229^, ^230^ Although HBOCs have been studied for decades and have undergone several generations, no HBOCs have yet been clinically approved. This is mainly due to side effects associated with HBOCs that have not been addressed, such as nephrotoxicity and elevated hypertension.^231^

**Oxygen‐producing nanocarriers**: Excess hydrogen peroxide (H_2_O_2_) exists in the hypoxic microenvironment of tumors, which greatly limits the efficacy of tumor therapy. Materials such as catalase (CAT)^232^, ^233^, ^234^ and manganese dioxide (MnO_2_)^235^, ^236^ can use H_2_O_2_ as a raw material to generate abundant oxygen, acting as oxygen generators to improve tumor oxygenation and existing therapeutic efficacy.^237^ Song designed a novel bionanoreactor to decompose tumor endogenous H_2_O_2_ and relieve tumor hypoxia by encapsulating CAT in tantalum oxide (TaOx) nanoshells, resulting in TaOx@Cat nanoparticles.^238^ Lalit Chudal encapsulated protoporphyrin IX in a liposome bilayer and then coated the lipid bilayer with MnO_2_ nanoparticles to improve the efficacy of photodynamic therapy under tumor hypoxia.^239^ Therefore, integrating CAT and MnO_2_ into bionanoreactors provides an attractive nanotechnological route to effectively enhance tumor therapy.

Over the years, nanomaterials have been used as delivery platforms and in vivo diagnostic carriers due to their unique nano properties and physical and chemical properties, and many achievements have been made. Blood vessels in tumor tissues are very different from those in normal tissues and have a higher density. There is a large gap between vascular endothelial cells, and the blood vessels of tumor tissue have an EPR effect; therefore, nanomaterials can target tumor tissue and improve the accumulation of drugs in tumor tissue. Compared with atmospheric hyperoxia and hyperbaric hyperoxia, which improve tumor pO_2_, the delivery of nanomaterials can more specifically target tumor tissues and improve tumor oxygenation.

### 4.4 Hyperthermia increases tumor oxygenation by increasing the intratumoral blood flow supply

Hyperthermia (HT) is defined as an increase in the body tissue temperature above the normal physiological range, approximately 39–45°C.^240^ In solid tumors, hyperthermia increases tumor blood flow and increases the tumor perfusion fraction in a temperature‐ and time‐dependent manner. Variations in blood circulation in tumors can create dramatic physiological changes, including enhanced vascular permeability, increased oxygenation, decreased interstitial fluid pressure, and restoration of normal physiological pH.^241^ In the 1970s, a study of the effect of 45°C isotonic lavages of the bladder on proliferative transitional cell carcinoma showed a clear anticancer effect of hyperthermia.^242^ HT therapy for cancer treatment has primarily focused on localized hyperthermia (i.e., heating the area surrounding the tumor), and existing clinical data suggest that the use of localized hyperthermia in combination with conventional radiotherapy may be valuable.^243^, ^244^ Other research groups have also investigated the application of systemic hyperthermia in antitumor therapy.^245^, ^246^, ^247^ From these studies, two main whole‐body hyperthermia regimens have emerged: (1) the traditional short‐duration hyperthermia regimen, that is, core body temperature rises to 41.88°C for 2 h; (2) a mild hypothermic long‐duration regimen, that is, core body temperature rises to 39.5‐40.8°C for 6 h or more. Sun demonstrated that the effect of HT (41°C, 30/45 min) on tumor oxygenation was different in different regions in a xenograft model. Overall, tumor oxygenation improved during and after heating, but the effect was transient.^248^ Thrall et al. treated 37 cases of spontaneous canine tumors with fractions of an equivalent total thermal dose of 30 CEM_43_T_90_ (a thermodosimetric measure, taking into account the 90th percentile temperature, not the median temperature of CEM_43_) divided weekly, and the animals received 1 or 3–4 divided treatments. Compared with baseline, the tumor PO₂ was significantly increased in both treatment groups.^249^

HT increases the local temperature of the tumor, accelerates the circulation, and increases oxygen saturation, thus increasing the oxygen content of the tumor, increasing the number of cells in the sensitive phase of the tumor, and reducing the number of hypoxic cells in the center of the tumor. No damage to the surrounding normal tissue has been observed. As a cancer treatment method, studies have shown that HT can improve the efficacy of conventional cancer treatments, especially radiotherapy.^250^ These studies suggest that further research on the antitumor effect of HT on tumor oxygenation enhancement is merited on a larger scale.

### 4.5 Carbogen respiration induces tumor hyperoxia

In the 1960s, Dusault explored the use of carbogen respiration in C3H breast tumor xenografts and found improvements. Studies have shown that carbogen improves blood flow and oxygenation more than pure oxygen because respiratory stimulation and vasodilation from carbon dioxide deliver more oxygen to the tumor.^251^ Other studies have also supported this conclusion.^252^, ^253^, ^254^ Carbogen respiration is defined as the inhalation of a 95% O_2_ + 5% CO_2_ mixture at standard atmospheric pressure.^255^ Inhalation of different CO_2_ concentrations showed different effects on the time course of tumor oxygenation changes. The perfusion recovered to its starting value immediately after the end of hyperoxia (pure oxygen), whereas with high concentrations of CO_2_, the perfusion remained elevated for at least 30 min. Higher inhaled CO_2_ concentrations (2.5% or 5%) prolonged the time to improvement in tumor perfusion compared with pure oxygen respiration.^256^ Carbogen respiration appears to have a greater effect on tumor oxygenation than respiration hyperoxia because carbogen induces vasodilation and increases blood flow to facilitate oxygen delivery.^191^ Nevertheless, the results of carbogen respiration were inconsistent, which may be due to the inconsistent timing of patients receiving carbogen respiration.

### 4.6 Tumor vascular normalization to improve tumor oxygenation

The concept that growing tumors have a rich vascular network first emerged through the observations of the well‐known scientist Rudolf Virchow.^257^ In 1939, Gordon and colleagues studied the relationship between tumor growth and blood supply in rabbit grafts, and they observed that tumor growth was accompanied by the rapid and extensive formation of new blood vessels.^258^ Therefore, antiangiogenic drugs have a synergistic antitumor effect in combination with radiotherapy and chemotherapy in clinical practice.^259^, ^260^, ^261^, ^262^, ^263^ However, hypoxia caused by the abnormal vascular state of tumors is the main cause of reduced radiotherapy and chemotherapy effectiveness, which contradicts the clinical facts. With the proposal of the “vascular normalization” theory, accumulating evidence has shown that antiangiogenic drugs normalize the overall function of tumor vessels before disrupting tumor vessels to improve tumor blood supply and increase the tissue oxygen content.^259^, ^264^ With the discovery of VEGF as a major driver of tumor angiogenesis, new therapies to inhibit VEGF activity have been studied.^265^ In 1993, it was reported that treatment with anti‐VEGF monoclonal antibodies resulted in a significant reduction in vessel density and a significant delay in tumor growth in nude mice carrying rhabdomyosarcoma, glioblastoma multiforme (GBM), and leiomyosarcoma xenografts.^266^ The efficacy of anti‐VEGF monotherapy in human solid tumors is generally less than satisfactory,^267^, ^268^ with only moderate objective response rates and meaningless survival differences in phase 3 clinical trials.^269^ In metastatic CRC, the overall response rate of patients receiving bevacizumab (a monoclonal antibody to VEGF) was 3.3%.^270^ Similarly, the response rate among patients with metastatic breast cancer treated with the drug alone was 6.7%.^271^ In contrast, the addition of bevacizumab to standard first‐line chemotherapy regimens significantly improved progression‐free survival.^272^, ^273^, ^274^ In conclusion, antiangiogenic drugs improve tumor blood supply and oxygen content, and this idea of vascular normalization provides a new direction for tumor treatment. Reasonable combinations of antiangiogenic drugs with radiotherapy and chemotherapy provide a feasible solution for tumor chemoradiotherapy resistance.

### 4.7 Promotion of oxygen accumulation via inhibition of tumor cell respiration

Inhibition of cellular respiration oxygen consumption provides a different type of intratumoral oxygen supply protocol to conserve endogenous oxygen and overcome hypoxia.^135^ Cellular respiration is the main process by which living cells consume oxygen. The antimalarial atovaquone is a ubiquitin analog that reduces the oxygen consumption rate (OCR) by inhibiting mitochondrial complex III and reducing intratumoral hypoxia.^275^ In addition, studies have shown that metformin (Met), a typical antidiabetic drug, also alleviates tumor hypoxia by reducing oxygen consumption by inhibiting complex I in the mitochondrial electron transport chain.^276^, ^277^, ^278^ Hypoxic tumor cells mainly use glucose to produce glycolytic energy and release lactic acid, which can fuel the oxidative metabolism of tumor cells. Therefore, targeting lactate can selectively kill hypoxic tumor cells.^279^ 3‐Bromopyruvate inhibits hexokinase‐II activity, thereby inhibiting mitochondrial respiration and glycolysis and reducing mitochondrial oxygen consumption and ATP production.^280^ Yu constructed a vesicular photodynamic therapy (PDT)‐specific PV‐TS nanoeconomizer, and an oxygen‐saver concept was proposed. PV‐TS can respond to tumor reduction conditions by releasing NO to inhibit cellular respiration, thereby reducing oxygen consumption and enabling biochemical redistribution of cellular oxygen resources to conserve oxygen.^281^ Therefore, inhibition of mitochondrial respiration to reduce tumor oxygen consumption has become an alternative strategy to alleviate the hypoxic microenvironment.

## 5 ANTITUMOR EFFECTS OF HYPEROXIA THERAPY

**FIGURE 3:**
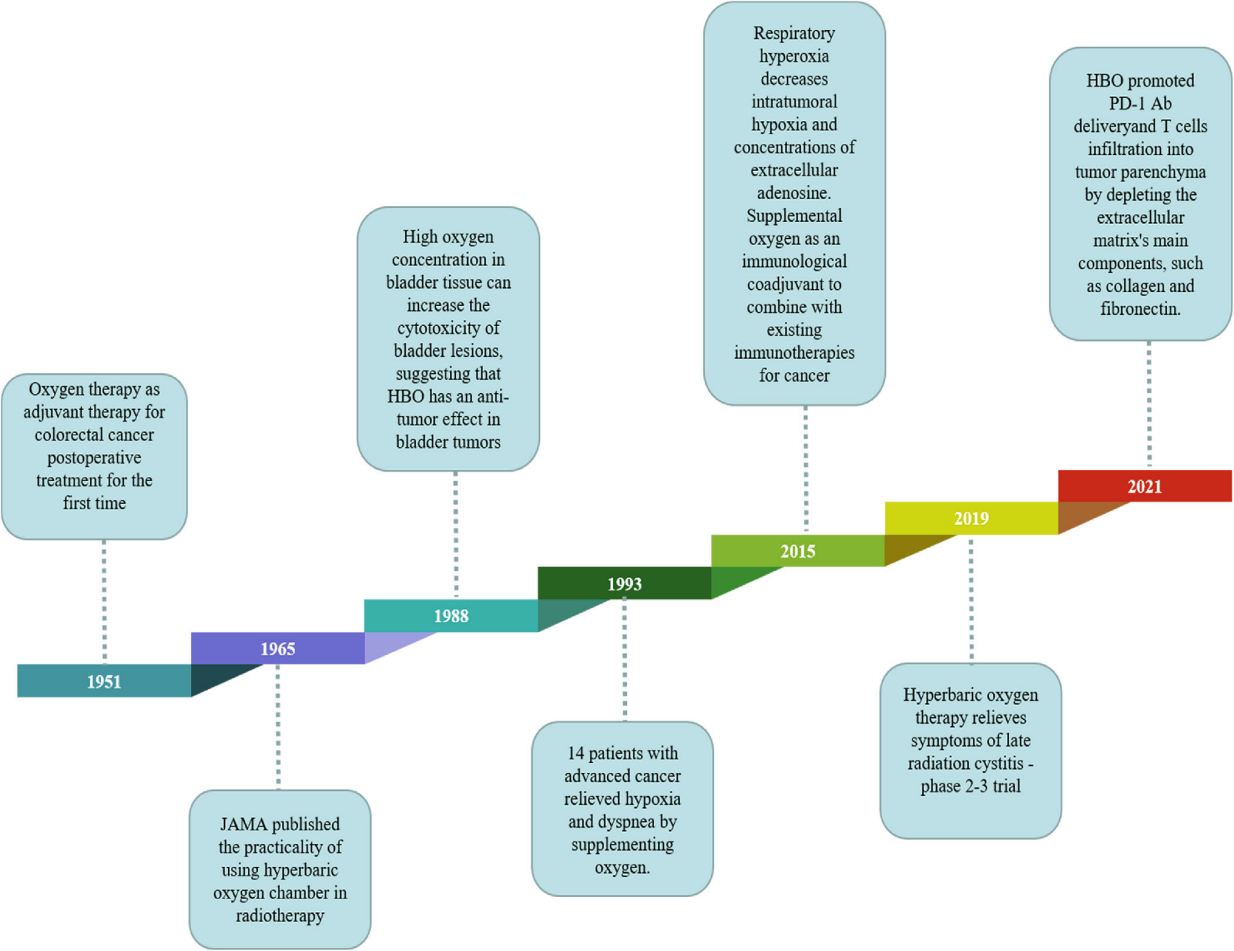
A timeline of respiratory hyperoxia discoveries and research history, highlighting key findings

Lung ventilation is the beginning of the respiratory function and the primary factor determining the efficiency of respiratory function.^282^, ^283^ Respiratory hyperoxia increases oxygen saturation in the lungs, increasing oxygen supply to the body.^284^ Oxygen therapy is one of the most widely used therapies in modern medicine and is a frequently performed intervention for critically ill patients.^18^, ^285^ In 1951, hyperoxia was used as adjuvant therapy in the postoperative treatment of rectal cancer and sigmoid colon cancer and was able to slow the occurrence of postoperative shock.^202^ In this section, the antitumor effects of hyperoxia therapy in the treatment of pulmonary tumors and non‐pulmonary tumors are the focus, and the landmark events in the application of hyperoxia for tumor treatment are outlined in Figure 3.

### 5.1 Hyperoxia therapy in pulmonary cancer

In 1956, Heston and Pratt began to study the role of oxygen in lung tumors, and the results showed that elevated oxygen uptake increased lung tumor initiation in mice.^286^ The application of early hyperoxia in tumor therapy is controversial, and Lindenschmidt et al. demonstrated that sustained exposure to 70% oxygen in mice inhibited the development of lung tumors.^287^, ^288^ Conversely, different groups have observed that respiration hyperoxia can act as a promoting stimulus to induce lung tumorigenesis of neuroendocrine tumors.^289^, ^290^, ^291^, ^292^, ^293^, ^294^ Initially, the effect of hyperoxia on cancer cells was poorly characterized. With the deepening of hyperoxia research and the understanding of the hypoxic TME, respiration hyperoxia was not thought to promote tumor development. Margaretten demonstrated that the effect of hyperoxia on lung tumors depends on the oxygen tolerance of lung tumors, and hyperoxia has a more pronounced inhibitory effect on oxygen‐sensitive cells.^295^ Witschi also provided an explanation for the role of hyperoxia in tumor development. Exposure to hyperoxia before or after drug administration usually promotes tumor development, and further exposure to hyperoxia for a period of time begins to suppress tumor development, but the specific mechanism has not been deeply explored.^296^ Liu demonstrated that intracellular metabolic reprogramming driven by the MYC/SLC1A5 axis is the main mechanism by which hyperoxia (60% O_2_, 6 h/day) inhibits lung cancer metastasis.^297^ The antitumor effect of atmospheric hyperoxia may be due to oxidative stress and increased apoptosis (BCL2‐associated X [Bax]/Bcl‐2 mRNA expression rate), which is associated with the mitogen‐activated protein kinase (MAPK) pathway.^298^, ^299^ In the early stage, the antitumor effect of hyperoxia therapy was mainly studied in lung tumors. With in‐depth studies, scientists have gradually explored the expanded antitumor effect of hyperoxia therapy by increasing the investigated tumor types.

### 5.2 Hyperoxia therapy in non‐pulmonary cancer

Improved tumor oxygenation also has a suppressive effect on non‐pulmonary tumors. Studies have shown that atmospheric and hyperbaric hyperoxia can reduce the hypoxic area of ​​tumors to varying degrees.^300^, ^301^, ^302^, ^303^ The EPO levels in hepatoblastoma HepG2 cells treated with 5% O_2_ and 1% O_2_ were 2.5 and 4 times higher than those in cells treated with 20% O_2_, respectively. In contrast, hyperoxia (40% O_2_) significantly inhibited EPO production.^304^ Hypoxia also overexpresses/activates STAT3, leading to tumor progression and drug resistance. Human ovarian cancer xenograft mice were exposed to HBO (100% oxygen, 2 ATM, 1.5 h) for 21 days. Mice exposed to HBO had significant reductions in tumor volume, but no effect on body weight was observed. After HBO exposure, STAT3 (Tyr 705) activation and cyclin‐D_1_ protein/mRNA levels were significantly reduced.^210^ Tumor cells and stromal cells were isolated, and the isolated cells had differential gene expression. HBO (2.5 bar, 100% O_2_, 90 min) had a significant inhibitory effect on tumor growth, and the MAPK pathway was significantly downregulated. An antiangiogenic effect was observed after intermittent HBO treatment.^305^ To date, several reviews have been published on the use of respiratory hyperoxia in cancer therapy.^306^, ^307^, ^308^, ^309^, ^310^

**FIGURE 4:**
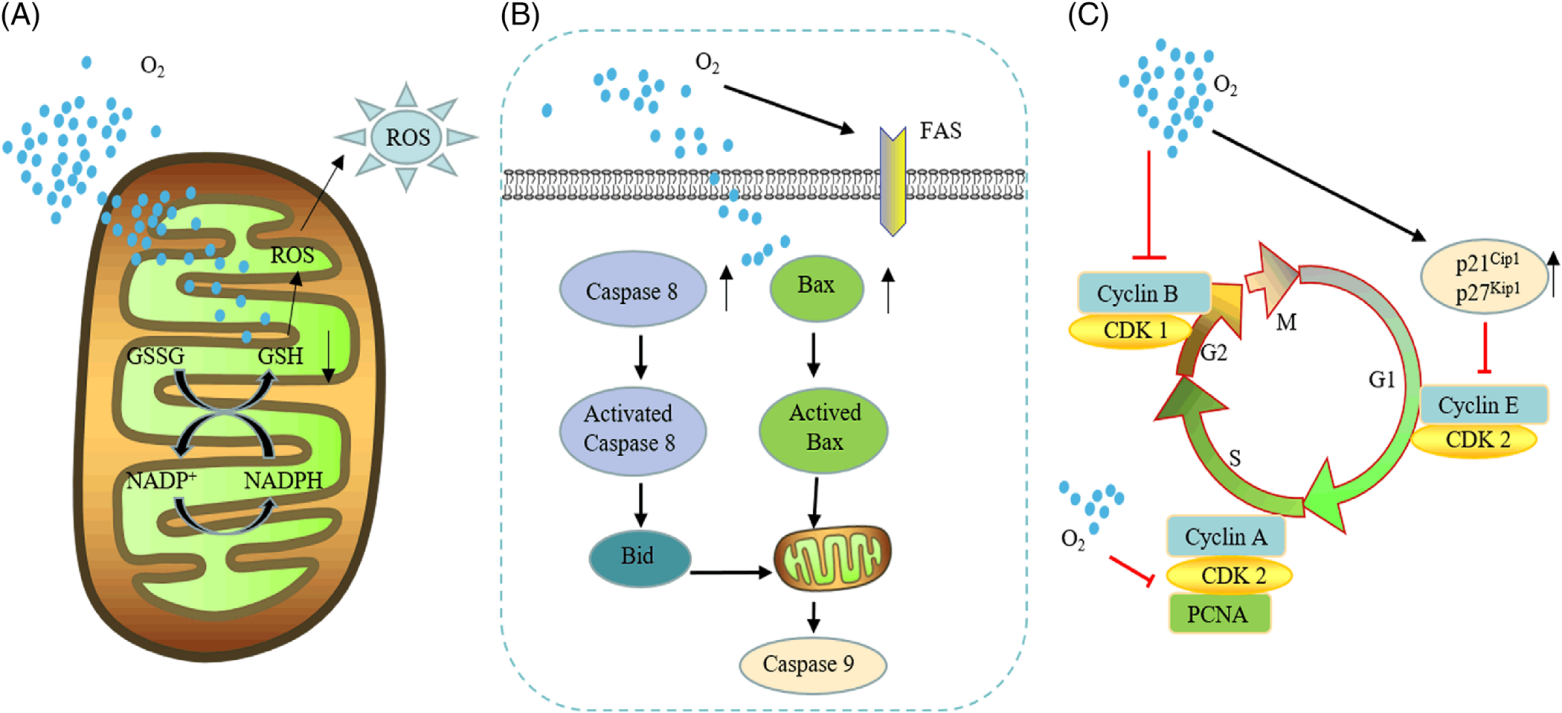
Antitumor effect of hyperoxia. Respiratory hyperoxia can through the following mechanisms. (A) Hyperoxia downregulates the concentration of glutathione in tumor cells, leading to intracellular oxidation‐reduction imbalance, excessive reactive oxygen species (ROS) production, and cell damage. (B) Hyperoxia promotes the increase of apoptosis‐related proteins Bax and Caspase 8, breaking mitochondria, and cell death. (C) The strong decrease in tumor cell proliferation under hyperoxia is associated with a marked inhibition of cell cycle progression, which is mainly characterized by the arrest in the G1, G2, and S phases. This G1/S phase arrest is associated with severe inhibition of cyclin‐dependent kinase 2 (CDK2) activity and DNA synthesis, and cell cycle progression from G1 to S is also inhibited. At the same time, hyperoxia induces cell arrest in the G2 phase by preventing the dimerization of cyclin B and the CDK1 complex and promotes cell apoptosis

The different responses of cancer cells and normal cells to hyperoxia also provide a principle of action for hyperoxia in cancer therapy. Little attention has been given to the differences in the way normal cells and cancer cells respond to changes in oxidative stress. The basic antioxidant defense level is usually abnormal in tumor cells. Glutathione (GSH), an important antioxidant in the body, can scavenge free radicals and prevent the induction of ROS damage.^311^ In normal cells, the GSH concentration is proportional to ambient oxygen tension. Compared with normal cells, tumor cells exhibited higher GSH concentrations under low oxygen tension but were unable to increase GSH with elevated oxygen tension. Tumor cells showed higher sensitivity to hyperoxia‐induced increases in ROS activity, while normal cells were not damaged by ROS due to elevated GSH.^312^ Similarly, Kim and co‐workers demonstrated that the reduced GSH/oxidized GSH (GSSG) ratio was significantly reduced after hyperoxia in both normal and cancer mouse groups. After 24 h of hyperoxia, ROS levels in cancer cells were significantly increased compared with those in normal cells. This result suggests that oxidative stress is more pronounced in cancer tissues exposed to hyperoxia than in normal tissues exposed to hyperoxia and that an increase in oxidative stress above the toxicity threshold leads to cancer cell death (Figure 4A).^313^

Apoptotic factors are also involved in the necrosis of tumor cells under the hyperoxia response, and the activation of hyperoxia‐induced apoptosis factors (Bax) can lead to cell death with or without loss of mitochondrial function (Figure 4B).^314^, ^315^ Hyperoxia also has inhibitory effects on the cell cycle. Hyperoxia exposure (95% O_2_, 40–64 h) induces cyclin‐dependent kinase 2 activity and acute inhibition of DNA synthesis. The S‐phase block also has different degrees of blockade in the G_1_ and G_2_ phases (Figure 4C).^316^, ^317^, ^318^, ^319^ Multiple reports have suggested that reduced oxygen tension can induce cancer growth by adapting malignant cells to hypoxia, producing antitumor immunity and maintaining and promoting cancer progression.^46^, ^52^, ^126^, ^320^ Hyperoxia can overcome tumor tissue proliferation^321^ and the issue of tumor development^322^, ^323^, ^324^ by alleviating the hypoxic environment, inducing glandular loss, causing DNA damage, and promoting mesenchymal‐to‐epithelial transition (MET).^325^

According to current studies, respiratory hyperoxia has inhibitory effects on lung tumors and other tumor types, with no significant difference in effect. Respiratory hyperoxia can alleviate tumor hypoxia and inhibit tumor progression through various mechanisms (Table 1). Although respiratory hyperoxia has been shown to be an effective way to eliminate tumor hypoxia, there are obvious limitations to oxygen transport and delivery far from blood vessels in the chaotic TME. Therefore, we recommend combining oxygen with other clinical treatment options. We believe that oxygen is an effective tool for improving radiotherapy and chemotherapy to maximize tumor rejection of current cancer therapies.

**TABLE 1:** Summary of antitumor effects of hyperoxia in cancer therapy

**Year**	**Tumor type**	**Type of hyperoxia therapy**	**Molecular basis**	**Reference**
1989	Hepatoblastoma	Atmospheric hyperoxia (40% O_2_)	Erythropoietin (EPO)	Ueno et al.^304^
1998	Breast cancer	Atmospheric hyperoxia (100% O_2_, 5 and 24 h)	Spermidine/spermine N1‐acetyltransferase (SSAT)	Chopra et al.^313^
2000	Breast cancer	Atmospheric hyperoxia (95% O_2_, 40 h)	Cdk2	Bilodeau et al.^316^
2002	Fibrosarcoma	Atmospheric Hyperoxia (95% O_2_)	Bax/Bak	Budinger et al.^315^
2009	Breast cancer	Hyperbaric oxygen (2 bar, 100% O_2_, 90 min)	Mesenchymal‐to‐epithelial transition (MET)	Ingrid Moen et al.^325^
2010	Ovarian cancer	Hyperbaric oxygen (2 bar, 100% O_2_, 90 min)	STAT3 and cyclin‐D_1_ protein	Selvendiran et al.^210^
2012	Breast cancer	Hyperbaric oxygen (2 bar, 100% O_2_, 90 min)	MAPK pathway	Moen et al.^305^
2017	Breast cancer	Hyperbaric oxygen (O_2_ > 97%, 2.5 ATM)	N‐cadherin, Axl, and type I collagen	Sletta et al.^209^
2017	Lung adenocarcinoma, Colorectal cancer	Atmospheric hyperoxia (95% O_2_)	Suppressor of morphogenesis of genitalia (SMG‐1) and p53	Resseguie et al.^326^
2018	Lung cancer	Atmospheric hyperoxia (95% O_2_ with normoxia cycle for 24 h)	MAPK pathway	Kim et al.^298^
2018	Breast cancer	Atmospheric hyperoxia (95% O_2_, 44 h)	Sirtuin 3	Pinterić et al.^327^
2020	Lymphoblastoma	Atmospheric hyperoxia (O_2_ > 60%, 24 h)	Caspase‐3	De Bels et al.^328^
2020	Triple negative breast cancer	Atmospheric hyperoxia (95% O_2_, 16 h)	Sirtuin 3	Podgorski et al.^329^
2022	Lung cancer	Atmospheric hyperoxia (60% O_2_)	MYC/SLC1A5	Liu et al.^297^

## 6 ADJUNCTIVE ROLE OF HYPEROXIA IN CANCER THERAPY

### 6.1 Application of hyperoxia in radiotherapy

**FIGURE 5:**
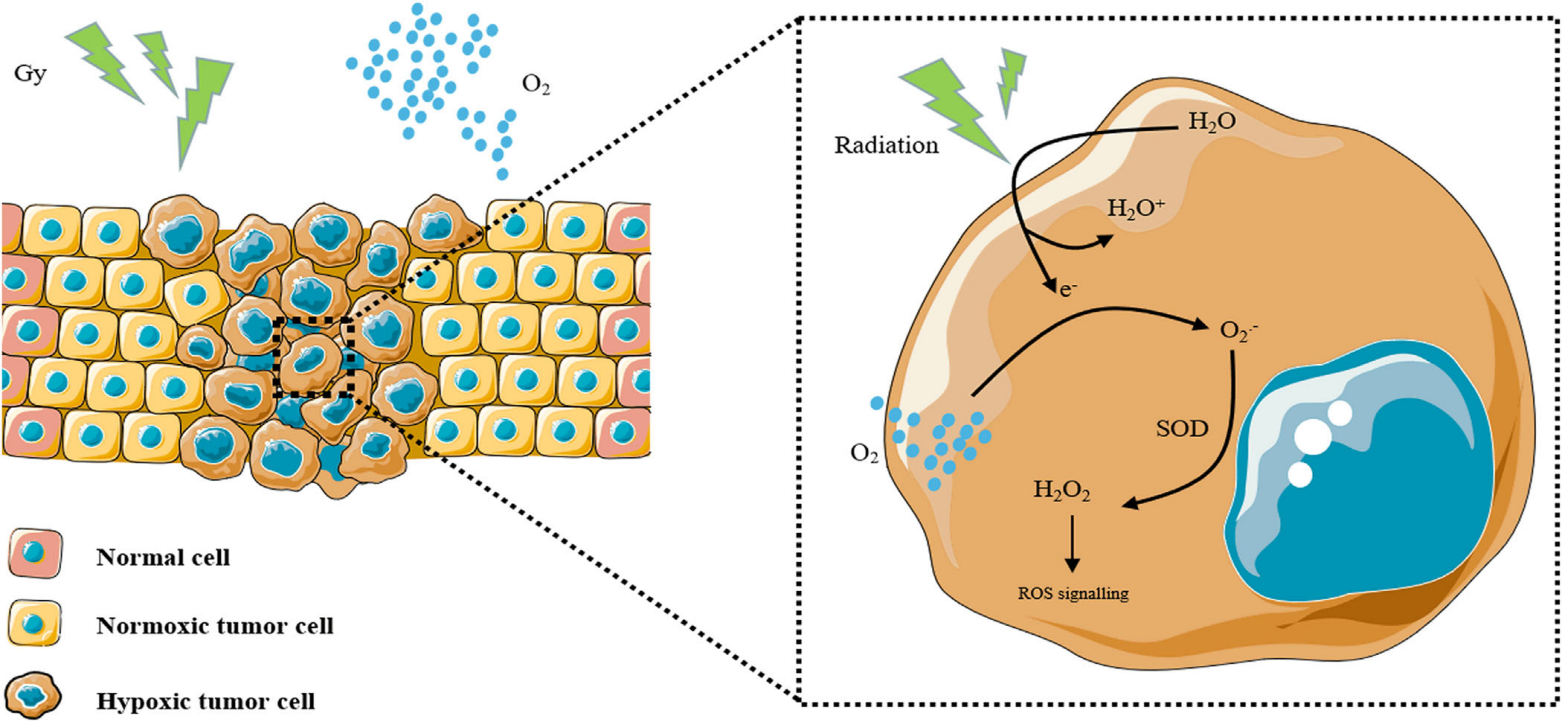
Schematic diagram of the antitumor effect of hyperoxia‐assisted radiation therapy. Radiation ionizes the tumor tissue water to generate electrons (e^–^), which need to be combined with oxygen (O_2_) to generate oxygen free radicals (O2^.−^). These radicals are oxidized by superoxide dismutase (SOD) to produce reactive oxygen species (H_2_O_2_). Hyperoxia promotes the antitumor effect of chemotherapy

Radiation therapy has been used since the late 19th century as a means of controlling tumor burden, both for curative and palliative purposes. Following the discovery of the German physicist Wilhelm Konrad Roentgen in 1895, X‐rays were used for diagnosis, and less than 3 years later, radiotherapy was used to treat cancer.^330^, ^331^ In 1909, Schwarz demonstrated in a simple experiment that if the skin was clamped to reduce blood flow to the arm, the skin's response to radiation was significantly reduced.^332^ However, it was not until 1953 that Gray and colleagues had an open‐label study showing that radiation sensitivity was dependent on oxygen concentration and that the effectiveness of X‐ray therapy might be enhanced if cancer patients were breathing oxygen while being irradiated.^333^ Just 2 years later, Thomlinson and Gray proposed that hypoxia may be central to human tumors and is a major culprit of radiotherapy resistance.^334^ These findings suggest that tumor cells near the oxygen diffusion limit exhibit improved survival under subnormal oxygen stress conditions, making them resistant to radiation therapy. There is increasing evidence that tumors with more hypoxic cells are poorly responsive to radiation therapy.^335^, ^336^, ^337^, ^338^ Results from earlier clinical studies confirmed the importance of oxygen content in the biological effects of radiation therapy.^339^, ^340^, ^341^ When radiation is absorbed by biological matter, highly reactive free radicals are produced, and these free radicals ultimately cause tissue damage. The free radicals produced in the target body are unstable, and the damage caused by free radicals is permanent and fixed until further reactions produce stable changes. However, if this process occurs under hypoxia or in the presence of reducing substances, the free radicals chemically return to their original form, and the damage is repaired (Figure 5). Therefore, under hypoxic conditions, the radiation dose would need to be increased two‐ or threefold to achieve the same level of damage.^342^

Radiobiology researchers have improved the efficacy of radiotherapy by reversing tumor hypoxia through various means, including the use of HBO therapy,^343^ nanoparticle oxygen platforms,^344^, ^345^ hypoxic cell sensitizers^346^, ^347^ and biological induction agents.^253^, ^348^ Molecular oxygen has long been recognized as a powerful regulator of cellular sensitivity to radiation.^211^ It has been reported that the biological effects of ionizing radiation are increased approximately 3‐fold when irradiation occurs under well‐oxygenated conditions compared to hypoxic conditions.^333^ Nordsmark applied hyperoxia (100% O_2_) and carbogen respiration (95% O_2_ + 5% CO_2_) to improve tumor oxygenation, and the results showed that both carbogen and oxygen inhalation increased tumor PO_2_ and radiation therapy effectiveness.^349^, ^350^ In mouse lung cancer, the combination of hyperoxia and radiation increased cell death and DNA damage compared to hyperoxia or radiation alone.^351^ HBOT, which uses oxygen as a therapeutic, dissolves oxygen in plasma and has been found to improve oxygen supply to hypoxic tumor cells, a treatment that has been used in conjunction with radiation therapy to treat malignancies.^352^ After HBO treatment, the elevated intratumoral oxygen tension is retained for a period of time, making it possible to enhance the tumor/normal tissue oxygenation ratio during radiotherapy.^353^, ^354^, ^355^ In the past decade, nanoradiosensitization has been a research hotspot. Nanoparticles generate oxygen in hypoxic regions of tumors through oxygen‐carrying/oxygenation without any oxygen toxicity to other tissues and organs.^356^ Recently, developed erythrocyte membrane‐encapsulated perfluorocarbon particles were shown to have fairly efficient oxygen loading and to efficiently deliver oxygen into solid tumors, facilitating radiotherapy‐based cancer therapy.^344^ Membrane vesicle‐encapsulated CAT alleviates tumor hypoxia‐enhanced radiotherapy.^357^

Fractionated radiotherapy is the irradiation mode adopted by most radiotherapy centers.^358^ Fractionated radiotherapy can transform cancer cells in quiescent and hypoxic states into proliferating cancer cells with high oxygen content during the rest period of radiotherapy, thereby increasing the sensitivity to radiation.^359^ In the phase I/II study, the hypoxic cell sensitizer misonidazole (MISO) was used as an adjunct to high‐fractional dose radiotherapy for unresectable stage III and IV oral, oropharyngeal, and hypopharyngeal squamous cell carcinoma.^360^ However, the study showed that MISO does not improve the efficacy of high fractional dose radiotherapy. However, this was a useful finding for future clinical trials using HBO or new hypoxic cell sensitizers to facilitate high‐fractional dose radiotherapy. Recently, it has been found that an ultrahigh dose rate of ionizing radiation has the advantage of protecting normal tissue and killing tumor cells, and this approach was named “FLASH radiotherapy”. FLASH radiotherapy was first proposed by Vozenin and co‐workers in 2014.^361^ FLASH radiotherapy applies radiation at an ultrahigh dose rate several orders of magnitude higher than the current clinical routine radiotherapy, which can make normal tissues resistant due to abnormal hypoxia while still maintaining local tumor control.^362^ The underlying mechanisms leading to the FLASH effect have not been fully elucidated, but a significant effect on oxygen tension and ROS production is currently the most valid hypothesis.^363^, ^364^ It is well known that oxygen plays a key role in the DNA damage caused by ionizing radiation.^365^ The fact that O_2_ is a powerful radiosensitizer has practical implications for cancer treatment with energetic heavy ions, such as carbon ions.^366^ Additional carbon ions have a synergistic advantage when using flash radiotherapy because heavy ions can generate an oxygen‐containing microenvironment around their orbits due to water ionization; that is, near the end of the carbon ion path (Bragg peak region), they can produce abundant oxygen.^367^ This suggests that flash radiotherapy using carbon ions, which are more toxic to the tumor, can significantly improve treatment.

Regarding clinical outcomes, several reports have demonstrated that irradiation occurring immediately after routine fractionated HBO has antitumor activity.^368^, ^369^, ^370^ Early research showed that HBO promoted the curative effect of radiotherapy. In recent years, the effect of HBO seems to focus on the prognosis of radiotherapy.^371^, ^372^ The prognostic efficacy and safety of HBOT for overt osteoradionecrosis of the mandible were validated in 12 university hospitals. Patients received 30 preoperative HBOTs for 90 min at 2.4 ATA or placebo treatments, followed by 10 postoperative HBO sessions or placebo. The primary outcome measure was the 1‐year recovery rate from osteoradionecrosis, but the results showed that patients with pronounced osteoradionecrosis of the mandible did not benefit from HBO.^373^ In 2012, a randomized controlled trial showed that HBOT was associated with improved outcomes in patients with post‐radiation tissue contusion following radiotherapy for the head, neck, anal, and rectal tissue cancers.^374^ A randomized phase III trial to examine the clinical benefit of HBO in patients with chronic bowel dysfunction after radiotherapy for pelvic malignancies found no patients with radiation‐induced chronic gastrointestinal symptoms, including rectal bleeding, who benefited from HBO therapy.^375^ Thus, the combination of HBO with radiotherapy appears to be an attractive strategy for cancer treatment, but as a method to improve the side effects of radiotherapy, the effect of HBO is not satisfactory.

In the past, hyperoxia therapy was considered to promote tumor proliferation and metastasis; thus, hyperoxia therapy was contraindicated. However, a large number of basic and clinical studies have found that hyperoxia therapy not only does not promote tumorigenesis but enhances the effect of radiotherapy. Hypoxia is an important reason for the poor effect of tumor radiotherapy. Under hyperoxia, the partial pressure of oxygen in tissues is more than 10 times that under normoxia, which can greatly improve the hypoxic state of tumors and enhance the effect of radiotherapy. On the other hand, when radiotherapy is used to treat malignant tumors, normal tissues will also be killed. Hyperoxia therapy can improve the blood supply to normal tissues to protect and promote the repair of normal tissues. In conclusion, hyperoxia is feasible and effective in the treatment of tumors, especially in adjuvant radiotherapy.

### 6.2 Application of hyperoxia in chemotherapy

Drug delivery and cellular uptake of drugs in hypoxic regions are known to be affected by hypoxia or associated acidity, resulting in the relative resistance of hypoxic tumor cells to chemotherapy.^376^, ^377^ Hypoxia is considered to be a major driver of multiple drug resistance (MDR) to chemotherapeutic drugs.^378^, ^379^ Glioma stem cells (GSCs) may be induced by dedifferentiation under hypoxic conditions to maintain the stemness of GSCs, which are maintained in chemoresistance. Hyperoxia inhibits the dedifferentiation process, promotes the differentiation of GSCs, and increases the sensitivity of glioma cells to chemotherapy.^14^ In a phase III trial conducted before 1977, HBO was shown to act synergistically with radiotherapy to improve locoregional control of head and neck tumors.^380^ In noncancerous hypoxic tissues, such as normal tissues after irradiation, HBO significantly increases oxygenation and promotes angiogenesis, ultimately leading to increased blood flow.^381^ If HBO also induces angiogenesis in hypoxic tumors, the resulting improvements in long‐term oxygen and nutrient delivery and chemotherapeutic drug delivery may lead to enhanced antitumor effects of chemotherapeutic drugs. Alternatively, this improved method of administration may promote tumor growth that is too fast to control with traditional chemotherapy. Alagoz proposed complete HBO treatment before the first chemotherapy treatment to maximize angiogenesis and chemosensitivity. Once angiogenesis is established, HBO treatment can be stopped, and chemotherapy can be initiated. The experimental results demonstrated that HBO increases blood vessel density in large tumors, such as in epithelial ovarian cancer, and mice treated with cisplatin and HBO showed significantly delayed tumor growth compared with mice treated with cisplatin alone. HBO alters the tumor vascular architecture by inducing angiogenesis, which may resolve the problem of cisplatin entry into tumor cells and lead to the successful treatment of well‐vascularized tumors.^382^ Additionally, intermittent normobaric hyperoxia (95% O_2_) can promote the toxic effects of carboplatin on lung tumors.^383^ It is generally believed that some chemotherapeutic drugs require oxygen to generate oxygen free radicals, which in turn induce cytotoxicity and promote the effect of antitumor drugs.^384^ An alginic acid solution containing calcium peroxide and CAT was dropped into a calcium chloride bath to form Ca2^+^‐crosslinked microcapsules. The cytotoxicity of DOX is promoted by increased ROS production.^385^ However, due to the pathologically elevated interstitial fluid pressure and dense extracellular matrix (ECM) in solid tumors, the transport of antitumor drugs in tumor tissues becomes difficult.^386^, ^387^, ^388^ HBO further enhances antitumor efficacy by increasing oxygen tension, reducing collagen deposition in the tumor ECM, promoting the penetration and accumulation of DOX liposomes into tumors, and increasing the sensitivity of tumor cells to DOX liposomes.^389^, ^390^ The hypoxic cell‐targeting drug tirapazamine promotes the toxicity of the PARP inhibitor olaparib/pazopanib by killing hypoxic cells.^344^

Several studies on the remission of hypoxic tumors in combination with various chemotherapeutic agents have shown increased mean survival time and/or decreased tumor growth/metastasis.^391^, ^392^ In a mouse model of bladder cancer treated with a combination of HBO and nimustine, there was a therapeutic effect on a subset of bladder tumors.^393^ GBM is a highly malignant primary brain tumor originating from the glial tissue of the central nervous system,^394^ and resistance to the chemotherapeutic drug temozolomide is commonly responsible for most disease recurrences.^395^, ^396^, ^397^ Experimental results showed that hyperoxia leads to dysregulation of protein folding, which in turn induces apoptosis mediated by the unfolded protein response. Hyperoxia may induce resensitization of chemoresistant GBM cells.^398^ The cytotoxicity of first‐line chemotherapeutics, such as cisplatin, carboplatin, DOX, paclitaxel, and 5‐fluorouracil, is enhanced in the presence of hyperoxia (Table 2).^383^, ^389^, ^399^, ^400^, ^401^, ^402^

**TABLE 2:** Summary of hyperoxia combined with chemotherapy in antitumor treatment

Year	**Tumor type**	**Type of hyperoxia therapy**	**Combination therapy**	**Reference**
1989	Breast cancer	Atmospheric hyperoxia (95% O_2_)	Doxorubicin	Mimnaugh et al.^407^
2002	GH3 prolactinoma	Carbogen inhalation (5% CO_2_+95% O_2_)	Ifosfamide	Rodrigues^401^
2005	Breast cancer	Hyperbaric hyperoxia (97.9% O_2_, 2.1% CO_2_, 2.4 ATA bar, 90 min)	Mefaram, Gemcitabine, and Paclitaxel	Granowitz^321^
2005	Transitional cell carcinoma	Atmospheric hyperoxia (95% O_2_)	Gemcitabine	Fechner et al.^377^
2007	Breast cancer	Hyperbaric hyperoxia (1.5 bar, 100% O_2_, 90 min)	5‐Fluorouracil	Raa^322^
2009	Breast cancer	Hyperbaric hyperoxia (100% O_2_, 2 bar, 90 min)	5‐Fluorouracil	Moen et al.^389^
2011	Ovarian cancer	Hyperbaric hyperoxia (100% O_2_, 2 ATM, 90 min)	Cisplatinum	Karuppaiyah et al.^210^
2012	Glioblastoma multiforme	Atmospheric hyperoxia (40% O_2_, 80% O_2_)	Temozolomide	Sun et al.^397^
2014	Glioblastoma multiforme	Atmospheric hyperoxia (40% O_2_)	Temozolomide	Lee et al.^398^
2017	Glioblastoma multiforme	Atmospheric hyperoxia (95% O_2_)	Temozolomide	Wang et al.^14^
2018	Lung cancer	Atmospheric hyperoxia (95% O_2_, 3 h/day)	Carboplatin	Lee et al.^383^
2022	Hepatocellular carcinoma	Hyperbaric hyperoxia (100% O_2_, 2.8 ATA, 120 min)	Teniposide	Li et al.^302^

Hypoxia is a broadly studied phenotype at the clinical and molecular levels and a definite focus in biomarker studies; thus, exploring oxygen‐sensitive molecules to identify the degree of tumor hypoxia is a feasible approach. In addition to HIF, PHD, VHL, VEGF, CA9, and the other proteins mentioned above that can sense oxygen concentration, lysine demethylase 3A (KDM3A) and lysine demethylase 5C (KDM5C) should be included. Hypoxia inhibited the activities of KDM3A and KDM5C and reduced ROS production and tumor cell apoptosis.^403^, ^404^ In addition to inducing the transcriptional activation of multiple genes, hypoxia is also involved in the regulation of miRNAs. Studies have reported that miR‐210 is a highly upregulated miRNA in hypoxic cells and may serve as an in vivo marker of tumor hypoxia.^405^, ^406^ In the face of expanding drug combinations and measurable phenotypes associated with chemotherapy, it is necessary to explore the interrelationships between different oxygen‐related markers and tumor interventions. Clinical research is increasingly focusing on personalized treatment and the identification of biomarkers to guide treatment decisions for individual patients.

### 6.3 Application of hyperoxia in immunotherapy

**FIGURE 6:**
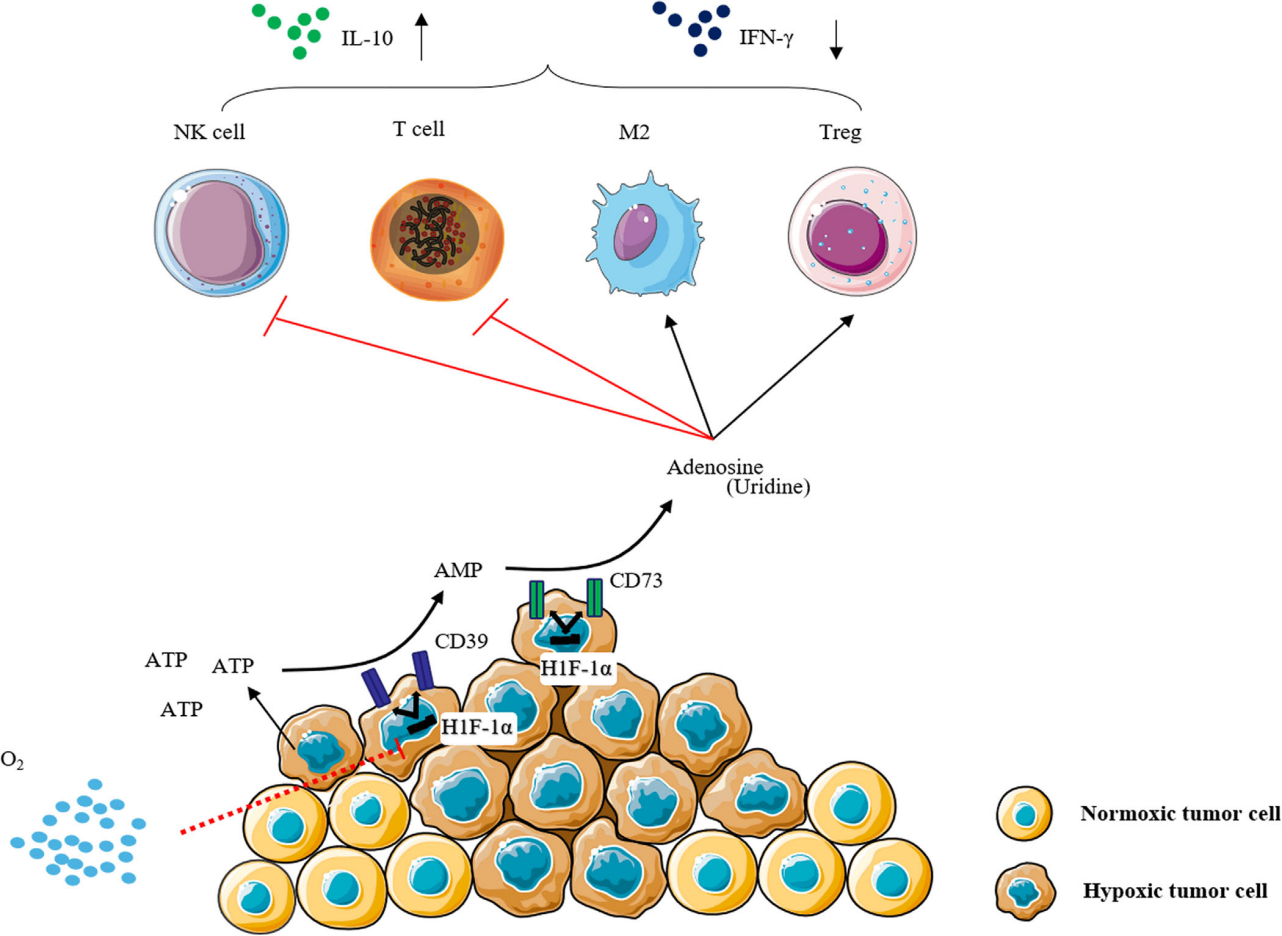
Hyperoxia reverses the hypoxic tumor microenvironment and promotes antitumor immunity. (A) Supplemental oxygen reduces the strength of downstream adenosine 2a (A2a)‐mediated tumor microenvironment (TME) immunosuppression by attenuating upstream tumor hypoxia. This in turn unleashes the antitumor activity of the otherwise suppressed T cells and natural killer (NK) cells, causing the tumor to regress

Another antitumor mechanism of hyperoxia may be to promote the revitalization of immunity. High concentrations of oxygen activate the anticancer effects of T cells and NK cells by deactivating the adenosine immunosuppressive microenvironment.^127^, ^206^, ^408^ In solid tumors, tumor hypoxia and HIF‐1α promote the upregulation of adenosine‐producing enzymes, such as CD39/CD73, which can dephosphorylate ATP to adenosine. High concentrations of adenosine impair the activation and function of T cells and NK cells and enhance the function of Tregs and the differentiation of M2 macrophages, resulting in strong immunosuppression. Increased tumor oxygenation may directly disrupt adenosine‐mediated immunosuppression by inhibiting the enzymatic activity of CD39 and CD73 to block adenosine production (Figure 6A).^206^ Before the discovery of immunonegative regulators and hypoxia/A2‐adenosine immunosuppression, it was unclear what factors in the TME prevented hundreds of millions of adoptively transferred T cells from destroying tumors in vivo. Stephen demonstrated that combined immunotherapy with respiratory hyperoxia can reduce tumor hypoxia and attenuate hypoxic/HIF‐1α/A2 adenosine immunosuppression, thereby exerting a powerful antitumor effect.^306^

**FIGURE 7:**
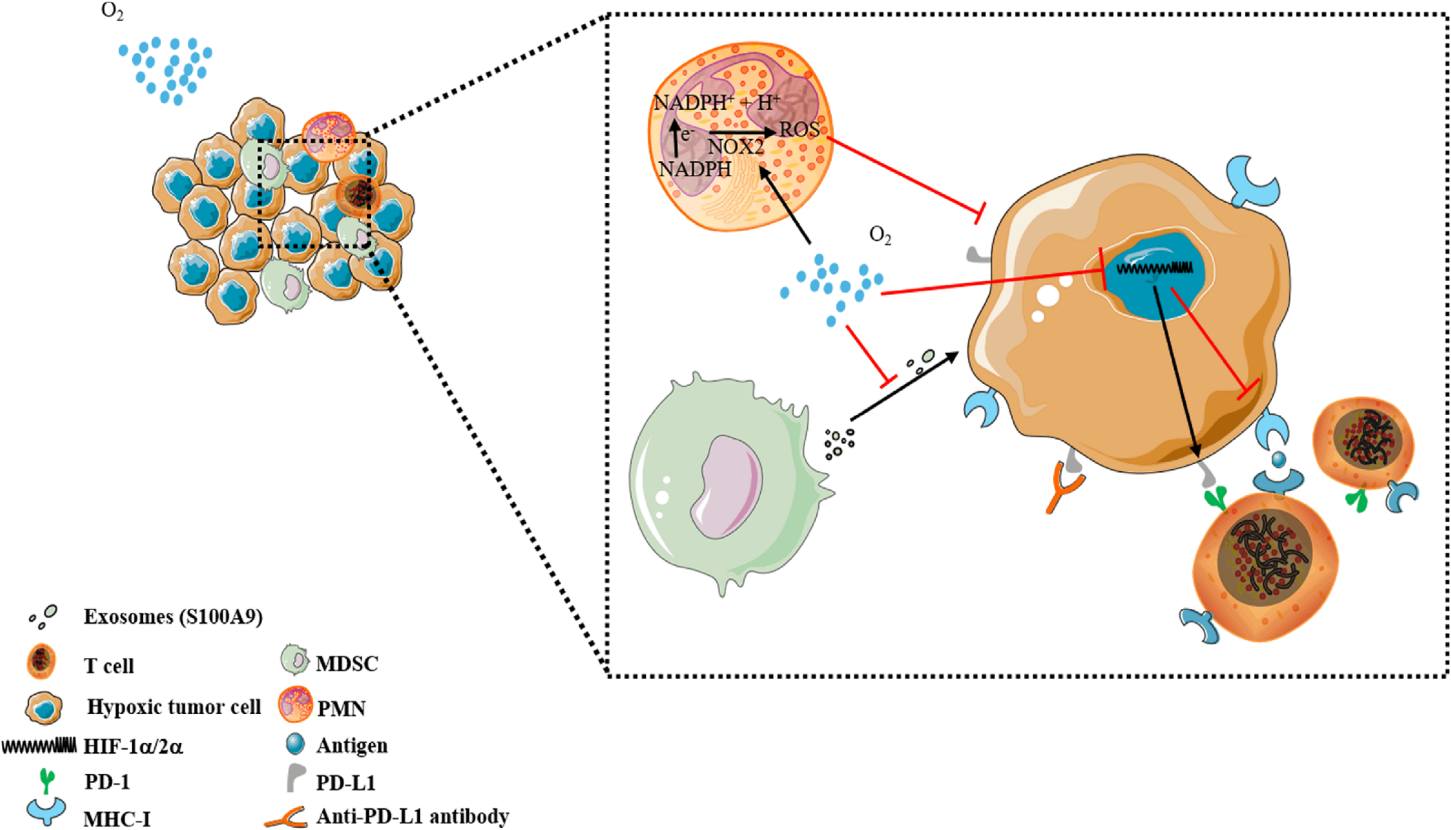
Hyperoxia promotes the expression of MCH‐1 and inhibits the expression of PDL‐1. PD‐1 binds to PD‐L1, which can transmit inhibitory signals and reduce the production of T lymphocytes. Hypoxia‐inducible factor (HIF) inhibits the in vivo immune effect by reducing MHC‐1 and increasing PD‐L1 expression in tumor cells. Hyperoxia has an antitumor effect by reversing tumor hypoxia and reducing PD‐L1 expression and can be applied in combination with an anti‐PD‐L1 antibody. Hyperoxia also promoted the upregulation of NOX_2_ in neutrophils, resulting in the increase of reactive oxygen species (ROS) and increased killing of tumor cells. Myeloid‐derived suppressor cells (MDSCs) can produce exosomes (mainly including S100A9) that promote the proliferation of tumor cells, and hyperoxia can reduce the production of exosomes by MDSCs and inhibit the proliferation of tumors

Polymorphonuclear neutrophils (PMNs) are thought to play an important role in cancer, but their role in tumors is unclear.^409^ HBO exposure may promote neutrophil clearance by macrophages.^410^ Mahiddine reported that hypoxia is a potent determinant of the tumor‐associated PMN phenotype and directly affects PMN‐tumor cell interactions. Hypoxia promotes the accumulation of PMNs inside tumors but then alters their phenotype in situ, limiting their ability to directly inhibit tumor growth. After the remission of hypoxia in respiratory hyperoxia, the tumor suppressor effect of PMNs was enhanced, resulting in a reduction in tumor burden despite reduced PMN infiltration.^411^ Myeloid‐derived suppressor cells (MDSCs) promote CRC tumor progression through multiple mechanisms. MDSC‐derived exosomes are considered to be intercellular messengers. Hypoxia induces granulocytic MDSCs (G‐MDSCs) to secrete more exosomes in a HIF‐1α‐dependent manner. Respiratory hyperoxia can inhibit the generation of G‐MDSC exosomes, reduce the stemness of CRC cells and inhibit the development of CRC (Figure 7).^412^

Enhancing tumor oxygenation as monotherapy can improve the tumor immune microenvironment; therefore, could combining hyperoxia and cancer immunotherapy improve the limitations of existing immunotherapies? Programmed death‐1 (PD‐1) blockade of the immune checkpoint has shown promising antitumor effects in a variety of cancer types.^413^ However, clinical studies have shown that only 30% and 40% of melanoma patients benefit from PD‐1 inhibitors, and PD‐1 blockade therapy is ineffective in most patients.^414^, ^415^, ^416^ Hypoxia, one of the most striking features of the TME, activates multiple signaling pathways that hinder the antitumor effects of various treatments, including immunotherapy.^417^ For example, HIF reduces the expression of major histocompatibility complex I (MHC I) while increasing the expression of PD‐L1, thereby reducing the tumor cell killing ability of cytotoxic T lymphocytes (CTLs),^418^, ^419^ while hyperoxia (60% O_2_) upregulates MHC expression, confirming that MHC antigen presentation regulation is oxygen‐dependent.^420^ The above findings suggest that alleviating the hypoxic TME may be an effective strategy to improve tumor immunotherapy represented by PD‐1 blockade (Figure 7).^421^ In triple‐negative breast cancer, 60% O_2_ can not only reduce the secretion of exosomes from MDSCs caused by hypoxia but can also inhibit the upregulation of PD‐L1 protein expression.^422^ HBO also promoted PD‐1 Ab delivery and T‐cell infiltration into the tumor parenchyma by depleting major components of the ECM, such as collagen and fibronectin. Furthermore, HBO disrupts hypoxia‐mediated immunosuppression and helps PD‐1 Ab trigger robust cytotoxic T lymphocyte responses and durable immune memory to suppress tumor recurrence.^423^, ^424^

Research has shown that an important component of hyperoxia therapy has been overlooked: the immune response. Normally, tumors grow faster than their blood supply, creating a hypoxic environment that stimulates cancer cells to produce a molecule called adenosine, which encourages antitumor T and NK cells to go into a dormant state. Given that respiratory hyperoxia inhibits the hypoxia‐adenosine pathway and promotes T cell infiltration into the tumor by depleting the dense ECM, we also expect hyperoxia to contribute to adoptive cell therapy, such as therapies that employ endogenous T cells and NK cells or genetically engineered T cells, NK cells and macrophages, by improving cell infiltration and functional maintenance in the malignant TME. Although the current studies have only been carried out in mouse models and this approach has not been tested in humans, they open up the possibility of using oxygen‐assisted immunotherapy to fight tumors.

### 6.4 Application of hyperoxia in PDT

PDT is a new therapeutic modality for the treatment of malignant tumors. It has been approved by the FDA for the palliative treatment of advanced lung and esophageal cancer.^425^, ^426^, ^427^ PDT utilizes certain dyes absorbed by the target tissue, such as photosensitizers. Photochemical activation of photosensitizers at tumors generates free radicals and/or ROS. This process requires oxygen molecules, and ROS can induce apoptosis or necrosis, microvascular damage, and immune responses.^428^, ^429^, ^430^ Therefore, the use of PDT for cancer treatment is based on the simultaneous participation of three factors, namely, retention of specific photosensitizers by tumor tissue, local illumination of lesions by visible light sources, and oxygen atoms.^431^ The efficiency of PDT is limited due to hypoxic regions and vascular aberrations in tumor tissue. Therefore, improving tumor tissue oxygenation can increase the oxygen content in tumor tissue and the content of singlet oxygen (the excited state of ordinary oxygen, which is a type of ROS), which can improve the efficiency of PDT.^432^ Studies have shown that the cytotoxicity of porphyrin and its related substances (photosensitizers) is mainly mediated by singlet oxygen, while hypoxic cells are less affected by porphyrin and light.^223^ The availability of molecular oxygen during PDT has been shown to have a profound effect on treatment outcomes.^433^, ^434^ Hypoxic cells are resistant to PDT treatment, and without oxygen, PDT has no cell‐killing effect.

Hjelde and colleagues investigated the effect of oxygen tension on photodynamic therapy toxicity in an established cell line.^435^ The authors demonstrated that hyperoxia did not affect the degree of toxicity of photodynamic therapy relative to normoxic conditions in vitro, in contrast to other experimental and clinical studies.^436^, ^437^, ^438^ They attributed the lack of an enhanced phototoxicity effect of hyperoxia to the fact that cell cultures may not have preexisting hypoxic cells or sufficient oxygen during photochemical reactions to compensate for oxygen depletion. The main reasons for the significant oxygen effects observed in other studies are the presence of hypoxic cells in solid tumors and the simultaneous occurrence of light and oxidation.^439^ Therefore, to take advantage of the oxygen effect, hyperoxic states must be ubiquitous. This is a very important detail because phototoxicity from PDT is dependent on both oxygen tension and photosensitizer concentration.^440^, ^441^ Nicola verified the effect of PDT under HBO on tumor tissue in the dorsal subcutaneous tissue of rats. Hematoporphyrin ester was used as a photosensitizer. Rats were pressurized to 3 ATM under 100% continuous oxygen ventilation in a specially designed hyperbaric chamber. The skin area above the tumor was photosensitized with a helium‐neon laser for 45 min. After 24 h, the tumor was excised for study. All PDT/HBO‐treated animals showed a marked reduction in the number of tumor cells and a large number of apoptotic and necrotic cells at the edges of the irradiated area compared to PDT control rats exposed to normal atmospheric conditions.^442^ Recently, several studies have shown that nanoparticles can also generate superoxide radicals while splitting water to produce oxygen, which can promote photodynamic therapy against tumors.^443^, ^444^, ^445^, ^446^ The tumor growth inhibition effect of PDT was promoted by loading photosensitizer and O_2_ into perfluorocarbon nanodroplets.^447^ In addition to generating ROS, oxygen can also assist PDT in decomposing collagen in the tumor ECM, thereby promoting the diffusion of oxygen and the penetration of photosensitizers deep into the tumor. This synergistic effect ultimately results in significantly improved therapeutic efficacy compared to photosensitizers alone at low laser power densities.^448^, ^449^ HBO combined with PDT can also significantly induce apoptosis and autophagy in human squamous cell carcinoma; therefore, HBO combined with PDT may be a promising approach for the treatment of human squamous cell carcinoma in the future.^450^

Maier evaluated the use of PDT under HBO versus normobaric conditions in patients with advanced esophageal cancer. All patients underwent photosensitization with hematoporphyrin derivatives, and of these patients, 14 received PDT alone, and 17 received PDT under HBO (2 ATA). Tumor length was reduced in both groups, and the tumor reduction was more pronounced in the PDT/HBO group. The 12‐month survival rate was 28.6% in the PDT group and 41.2% in the PDT/HBO group.^451^ Two clinical pilot studies demonstrated that combined PDT/HBO is a new and feasible therapy despite the small number of patients in the study. Combined PDT/HBO effectively and rapidly reduces intraluminal tumor burden and helps regulate further treatment procedures in patients.^452^, ^453^ Additionally, in the clinical treatment of advanced pleural malignant mesothelioma, investigators demonstrated that additional PDT/HBO is a safe and technically feasible approach (Table 3).^454^, ^455^ Currently, most PDT research focuses on nanomaterials and traditional PDT‐based nanotechnology to facilitate photosensitizer delivery and absorption while improving oxygenation in tumor tissue.^456^, ^457^, ^458^

**TABLE 3:** Clinical studies of hyperoxia combined with photodynamic therapy

**Year**	**Tumor type**	**Type of hyperoxia therapy**	**Progression**	**Result**	**Reference**
2000	Esophageal and cardia Cancer	Hyperbaric hyperoxia (100% O_2_, 2 ATA)	Clinical pilot study	There were significant differences in dysphagia score (*p* = 0.0064) and tumor length (*p* = 0.0002) in the PDT/HBO group. The median overall survival time was 7 months in the PDT group and 12 months in the PDT/HBO group (*p* = 0.0098).	Maier et al.^437^
2000	Esophageal cancer	Hyperbaric hyperoxia (100% O_2_, 2 ATA)	Clinical pilot study	Tumor length was reduced in both groups, with a significant difference in the PDT/HBO group (P = 0.002). Kaplan‒Meier analysis showed that the median overall survival in the PDT group and PDT/HBO group was 7.0 months and 12 months, respectively. The 12‐month survival rate was 28.6% in the PDT group and 41.2% in the PDT/HBO group (*p* = 0.059).	Maier et al.^451^
2001	Lung cancer	Hyperbaric hyperoxia (100% O_2_, 2 ATA)	Clinical pilot study	Tumor stenosis was significantly reduced (*p* < 0.05), and Karnofsky's performance was improved (*p* < 0.05) at 1 and 4 weeks after PDT/HBO treatment. No treatment‐related complications were found.	Tomaselli et al.^452^
2001	Malignant bronchostenosis	Hyperbaric hyperoxia (100% O_2_, 2 ATA)	Clinical pilot study	At 1 week and 4 weeks after PDT/HBO treatment, tumor stenosis was significantly reduced (*p* < 0.05), and Karnofsky's performance status was significantly improved (*p* < 0.05). There were no treatment‐related complications.	Tomaselli et al.^453^
2004	Malignant pleural stromal tumor	Hyperbaric hyperoxia (100% O_2_, 2 ATA)	Clinical pilot study	CT scans showed focal tumor regeneration after 6 months in 10/12 patients in the non‐PDT/HBO group. However, in the PDT/HBO group, only 9/22 tumors regenerated at the 6‐month follow‐up. Survival analysis showed a significant advantage in the PDT/HBO group (*p* = 0.0179).	Matzi et al.^454^
2004	Malignant pleural stromal tumor	Hyperbaric hyperoxia (100% O_2_, 2 ATA)	Clinical pilot study	During the 6‐month follow‐up, 8/11 patients in the non‐PDT/HBO group had focal tumor regrowth. In the PDT/HB group, 4/14 tumors regenerated. In the non‐PDT/HBO group, 9/11 patients died due to local tumor progression and distant metastasis, and 10/14 patients in the PDT group died due to local tumor progression and/or distant metastasis.	Matzi et al.^455^

Although hyperoxia can improve the efficacy of PDT treatment, HBO seems to be more effective for PDT. Study results indicate that HBO can promote the efficacy of PDT in addition to increasing blood pO_2_, but the specific mechanism remains to be explored. On the other hand, due to the variety of photosensitizers, the combination of hyperoxia therapy and photosensitizers that can better promote PDT requires more basic and clinical research. Oxygen supplementation can solve the problem of PDT resistance caused by tumor hypoxia, but the clinical application of hyperoxia combined with photodynamic therapy still needs further research.

## 7 FUTURE DIRECTIONS AND DISCUSSIONS

Cancer cells have a complex relationship with oxygen, and for decades, research has been conducted to investigate whether oxygen helps or harms cancer. Tumors that grow in hypoxic environments are known to be more metastatic and more lethal to patients. Tumors are initially susceptible to chemotherapy and radiotherapy, and advanced cancers maintain growth as cells adapt to a hypoxic microenvironment.^459^ Hypoxia severely hinders various anticancer strategies and is also a major driver of the development of tumor resistance, invasion, and metastasis.^460^ Tumor hypoxia has prompted the design of new approaches to fight cancer.

Theoretically, the application of hyperoxia therapy in tumor therapy is justified for the following reasons: improving oxygenation can remove hypoxia‐stimulated vascular malformations and degrade the dense ECM of tumor cells, improving drug delivery to hypoxic areas of tumors. The improvement in oxygenation causes cells to enter a proliferative phase, which makes them sensitive to radiation therapy and certain chemotherapeutic agents. Improved oxygenation also recruits immune cells to the tumor or enhances the antitumor ability of immune cells. With these facts in mind, although the antitumor effect of hyperoxia therapy is weak, it may improve the effectiveness of some therapies and be beneficial as an adjuvant in cancer treatment.

There are still many difficulties in the clinical application of hyperoxia therapy. Oxygen is breathed into the blood to provide nutrients for cells throughout the body. Cancer cells also need oxygen to survive. As earlier studies have shown, respiratory hyperoxia in the early stages of tumorigenesis accelerates tumor development by promoting angiogenesis.^289^, ^461^ This is the underlying theory of hyperoxia therapy as an adjunct therapy. Tumors thrive in the absence of oxygen and resist treatment; therefore, increasing oxygen supply to the tumor can return tumor cells to their normal state and thus resensitize them to existing therapies. Currently, hyperoxia is often used as adjunctive therapy for patients with advanced cancer, which reduces the antitumor efficacy of hyperoxia. The timing of hyperoxia intervention is the first problem to be solved. Another obstacle to the clinical application of hyperoxia therapy is the oxygen concentration and use time. Oxygen poisoning occurs with excess oxygen concentrations and oxygen inhalation times. Intermittent oxygen inhalation is recommended, and the patient treatment time is relatively flexible; thus, it is relatively easy to achieve clinical application. Several important questions remain unanswered about the mechanism of hyperoxia in tumor therapy and how to target hypoxia pathways in specific disease states. Every tissue, disease state, and individual has an optimal oxygen level. Mismatches in oxygen supply and demand can lead to pathological changes, and much remains unknown about the cellular response to hyperoxia. We should scientifically examine the hyperoxia therapy strategy and recognize the synergistic effect of hyperoxia therapy and existing tumor treatment methods. Isolated use of hyperoxia therapy to some extent restricts the further promotion of hyperoxia therapy in clinical practice. Therefore, hyperoxia therapy as a tumor treatment needs to be further validated.

At present, researchers seem to be highly interested in the effect of hyperoxia on immunity. Hyperoxia can recruit immune cells, such as T and NK cells, but its specific mechanism has not been fully studied. Why can hyperoxia recruit immune cells? One of the most important and basic effects of hyperoxia is to activate ROS, which kills tumor cells by damaging mitochondria. What is the connection with immunity? Although a large number of studies have verified the immune activation effect of hyperoxia, research on the antitumor effect of hyperoxia is limited. Therefore, the combination of hyperoxia therapy with existing immunotherapy can open up new treatment pathways and will need further exploration.

## AUTHOR CONTRIBUTIONS

Y.Z. and J.H.W. conceptualized this review and drafted the paper. Y.Z., K.L. and Q.Y.H. produced the figures and tables. J.H.W., X.S.G. and C.P.J. revised the paper. All authors have read and approved the article.

## CONFLICT OF INTEREST

The authors declare that they have no conflict of interest.

## ETHICS STATEMENT

Not applicable.

## Data Availability

Not appliable.
